# Detection of Protein and Metabolites in Cancer Analyses by MALDI 2000–2025

**DOI:** 10.3390/cancers17213524

**Published:** 2025-10-31

**Authors:** Dorota Bartusik-Aebisher, Daniel Roshan Justin Raj, David Aebisher

**Affiliations:** 1 Department of Biochemistry and General Chemistry, Medical College of The Rzeszów University, 35-310 Rzeszów, Poland; dbartusikaebisher@ur.edu.pl; 2 English Division Science Club, Medical College of The Rzeszów University, 35-310 Rzeszów, Poland; dj135639@stud.ur.edu.pl; 3 Department of Photomedicine and Physical Chemistry, Medical College of The Rzeszów University, 35-310 Rzeszów, Poland

**Keywords:** matrix-assisted laser desorption/ionization (MALDI), imaging mass spectrometry, cancer metabolites, metabolic biomarker, metabolomics, tumor microenvironment

## Abstract

**Simple Summary:**

Cancer continues to remain as one of the major causes of death around the world, for which early detection is essential in improving survival rates. This article focuses on how matrix-assisted laser desorption/ionization has revolutionized the way scientists study cancer metabolism over the past 25 years. The unique chemical changes in tissues can be identified with MALDI-MSI through the visualization of molecules whilst preserving spatial context. By gathering findings from two and a half decades of research, this review highlights the discoveries of diagnostic and prognostic markers and strengthens the connection between laboratory-based metabolomics and clinical care.

**Abstract:**

Cancer metabolomics has become a powerful way of understanding tumor biology, identifying biomarkers and metabolites, and helping precision oncology. Matrix-assisted laser desorption/ionization mass spectrometry (MALDI-MS), among many other analytical platforms, has gained popularity over the past two and a half decades due to its unique ability of directly analyzing metabolites in tissue with spatial resolution. This review will study 2000–2025 MALDI-based strategies for cancer metabolite detection, spanning from early proof-of-concept protein profiling to the development of high-resolution MALDI-MS imaging (MALDI-MSI), which is capable of mapping thousands of metabolites at near single-cell resolution. Its applications include the differentiation of tumor versus normal tissue, discovery of stage and subtype specific biomarkers, mapping of metabolic heterogeneity, and the visualization of drug metabolism in situ. Breakthrough technological milestones, such as the advanced matrices, on-tissue derivatization, MALDI-2 post-ionization, and the integration with Orbitrap or Fourier-transform ion cyclotron resonance (FT-ICR) platforms, have significantly improved the overall sensitivity, metabolite coverage, and spatial fidelity. Clinically, MALDI-MS has shown its purpose in breast, prostate, colorectal, lung, and liver cancers by providing metabolic fingerprints that are linked to tumor microenvironments, hypoxia, and therapeutic response. However, challenges such as the inclusion of matrix interface with low-mass metabolites, limited quantitation, ion suppression, and the lack of standardized procedures do not yet allow for the transition from translation to routine diagnostics. Even with these hurdles, the future of MALDI-MS in oncology remains in a good position with major advancements in multimodal imaging, machine learning-based data integration, portable sampling devices, and clinical validation studies that are pushing the field towards precision treatment.

## 1. Introduction

### 1.1. Cancer Metabolism

**Figure 1:**
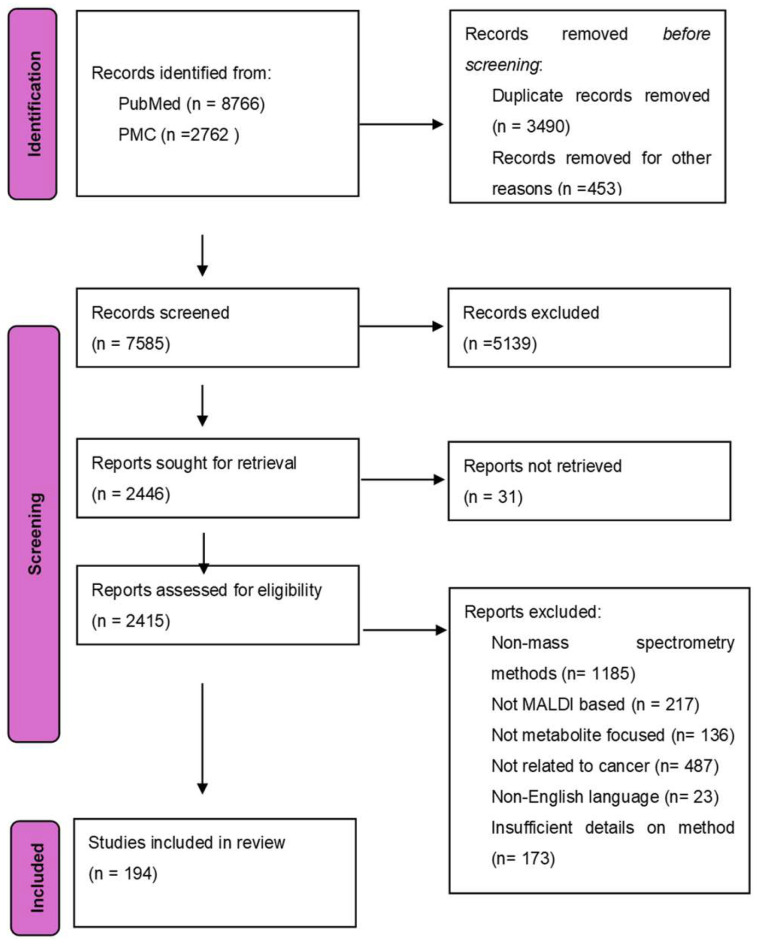
Preferred Reporting Items for Systematic Reviews and Meta-Analyses (PRISMA) flow diagram of the study selection for the review on MALDI-based cancer metabolite detection. Records were identified from PubMed (n = 8766) and PMC (n = 2762). After the removal of duplicates (n = 3490) and irrelevant records (n = 453), 7585 records were screened. Of which, 5139 were excluded at the screening stage, and 31 full-text articles could not be retrieved. Among 2415 articles assessed for eligibility, 2221 reports were excluded due to non-mass spectrometry methods (n = 1185), not MALDI-based (n = 217), not metabolite-focused (n = 136), lack of cancer relevance (n = 487), non-English language (n = 23), or insufficient methodological details (n = 173). A total of 194 studies were included in the final review.

Cancer is a disease that is identified by the unrestricted growth of cells and its ability to spread to other parts of the body. It is the leading cause of death in people aged younger than 85 years [1]. Despite the great strides taken in the world of cancer treatment till now, the disease is still without cures and has certain treatments that may only help cure certain cancers. Cancer cells are highly proliferative, multiplying from one aberrant cell to nearly 10^9^ cells [2], which is the average number of cells in a tumor of 1 cm in diameter. As cancer tumors develop, cancer cells meet and acclimatize to the many different metabolic stresses the acidic and hypoxic tumor microenvironment has to offer [3]. One of the most important traits of cancer cells is their ability to alter their metabolism to support the elevated energy demand and their malice in poor nutrient surroundings, such as a lack of oxygen. Unlike normal cells that rely on oxidative phosphorylation, cancer cells use aerobic glycolysis to produce energy, commonly referred to as the Warburg effect [4]. A PRISMA flow diagram of the study selection for the review on MALDI-based cancer metabolite detection is presented in Figure 1.

### 1.2. Importance of Metabolite Detection in Cancer

The diagnosis and treatment of cancer is complicated due to its various types, stages, and advanced biological mutations. An early detection of cancer is related to greater survival rates and thus is key in improving patient outcomes. Long-term survival rate drastically decreases as the cancer progresses, whereas patients diagnosed with stage IA cancer have a much higher survival rate [5]. Metabolite detection is crucial in both research and clinical practice because it gives functional insights on how cancer cells grow, survive, and spread. The detailed study of metabolites (metabolomics) is an excellent approach that enables the discovery of altered metabolic characteristics that can differentiate cancerous tissue from normal tissue [6]. For example, in breast cancer, metabolite detection has found distinct metabolic fingerprints, in addition to lipid, amino acid, and metabolism energy shifts, which all help in the early detection and diagnosis of cancer [7]. Metabolite detection is also able to differentiate cancer stages, for example, distinguishing between stage III and IV in pancreatic cancer or tumor types in ovarian and colorectal cancers based on serum or tissue metabolite patterns [8].

### 1.3. Role of Mass Spectrometry

Mass spectrometry is rooted in the study of metabolites and has shown to be the foremost technology in endogenous metabolite detection. It plays an important role in metabolite detection as it has the potential to precisely measure the mass-to-charge ratio of molecules (*m*/*z*). This makes the identification of a wide range of molecules, from simple amino acids to complex lipids and carbohydrates, possible. Metabolite detection performed with mass spectrometry is key in the field of research related to biomarker identification, drug target discovery, and the understanding of various disease mechanisms. It has been very commonly used in neonatal screening, with millions of infants and newborns tested via mass spectrometry to detect inborn metabolic disorders each year [9]. The adoption of soft ionization techniques, in particular matrix-assisted laser desorption ionization (MALDI), has pushed MS forward in metabolite detection [10]. MALDI, being a separation-free method, allows better metabolite coverage including volatile, non-volatile, ionizable, and spatially localized metabolites. It can provide straightforward spectra and ionization by producing single-charged ions, which makes spectral interpretation easier. This clear and smooth output reduces complexity and spectra noise, thereby enhancing detection clarity for metabolites.

### 1.4. Why MALDI?

Traditionally, electrospray ionization (ESI) in combination with liquid chromatography-mass spectrometry (LC-MS) has been the most popular technique used for cancer metabolomics. It is a soft ionization technique that ionizes analytes directly from liquid-phase into gas-phase ions, usually after the chromatographic separation, which is ideal for the detection of polar and semi-polar metabolites, such as amino acids, nucleotides, and other small organic acids. It has a very broad metabolite coverage, which is excellent for usually hard-to-detect hydrophobic metabolites. However, in comparison with MALDI, it is destructive and damages the integrity of the sample being analyzed. MALDI is also more time efficient as each laser shot is able to give a full mass spectrum within seconds, where LC can take up to 10–60 min per sample.

Between 2000 and 2025, MALDI has become a pivotal technique used in cancer metabolite detection, particularly by using mass spectrometry imaging (MSI) to visualize the distribution of metabolites within affected tissues. In spatial metabolomics, it showed the differentiation of metabolic heterogeneity on a tissue and cellular level in various cancers. It showed this at pixel resolutions ranging from sub-micrometer levels to 10 μm when using advanced methods like MALDI-2 and atmospheric pressure methods [11]. In prostate cancer, MALDI-FT-ICR-MS imaging using enhanced matrix deposition showed methods (MCAEF) found over 1000 metabolites, including lipids and small molecules. Many of these metabolites showed differential localization between cancerous and non-cancerous regions [12]. In lung cancer, high-throughput serum screening using MALDI and machine learning identified 13 metabolite features that clearly differentiated between patients, six of which were intact metabolites [13]. In liver metastasis, MALDI-MSI distinguished elevated lipid species (e.g., phosphatidylcholines and sphingomyelins) found in metastatic lesions from normal tissue [14].

By examining 26 years of MALDI’s scientific progress, this article aims to trace the pathway of MALDI from a regular analytical tool to being a cornerstone in cancer metabolite detection and its role in explaining cancer metabolism and advancing precision oncology.

## 2. MALDI Technology Overview

### 2.1. Fundamentals of MALDI

Matrix-assisted laser desorption/ionization (MALDI) is an ionization technique that utilizes a matrix that absorbs laser energy to produce ions from large molecules with little to no fragmentation [15]. In 1985, Franz Hillenkamp and Michael Karas put forth the concept that when non-UV-absorbing analytes (e.g., alanine) are mixed with UV-absorbing amino acids (e.g., tryptophan) under pulsed UV laser irradiation, it gives way for the ionization of the analytes and enables the ionization of biomolecules such as peptides (e.g., melittin, ~2843 Da). MALDI’s remarkable ability to show very high sensitivity even at sub-picomolar levels, its impressive tolerance to contaminants, and its high mass accuracy all further enhance its usability [16]. MALDI has also been used in a wide range of proteomics and clinical diagnostics (e.g., bacterial identification via matrix-assisted laser desorption ionization-time of flight (MALDI-TOF)), polymer analysis, and biomolecule characterization [17]. Key milestones such as the soft ionization of large biomolecules successfully ionized proteins up to ~34 kDa (carboxypeptidase A), which was a major early breakthrough, for which the developer, Koichi Tanaka, was awarded the 2002 Nobel Prize in Chemistry.

### 2.2. Ionization Process and Matrix Types

The current best-accepted model in MALDI is the two-step ionization process, which consists of primary and secondary ionization. Primary ionization occurs by cluster ionization or photochemical processes, during or soon after the laser pulse. Secondary ionization involves ion and molecule reactions within the expanding desorption plume, which leads to charge transfer and final analyte ion formation [18]. Other ionization mechanisms also exist, such as cluster ionization (CI), photochemical ionization (PI), and thermal proton transfer models [19], but the two-step model that combines primary and secondary ionization is the most commonly used. A good matrix in MALDI must be able to absorb laser energy, be vacuum stable, and facilitate proton transfer without damaging analyte molecules. Some commonly used matrices are CHCA (cyano-4-hydroxycinnamic acid) which is ideal for peptides and small proteins, sinapinic acid (3,5-dimethoxy-4-hydroxycinnamic acid) which is used for larger proteins and high mass analytes, and DHB (2,5-dihydroxybenzoic acid) which is often used for peptides, glycans, and in positive-ion mode MALDI imaging [20]. There are also some matrices that are up and coming, such as nanoparticle-based matrices like iron oxide, silver, and gold nanoparticles, and liquid crystalline and nanocrystalline matrices that are particularly beneficial for lipid analysis and small molecules.

The laser resolution is a crucial factor that influences spatial precision, sensitivity, and molecular coverage in metabolite imaging and detection. It usually ranges from 200 μm to around 10–20 μm in subcellular MALDI imaging systems. The matrix crystal size, uniformity following sample preparation, and ion diffusion within the tissue section, along with the diameter of the laser spot, are factors that affect laser resolution. Sample preparation is also an important determinant of data quality, interpretability, and reproducibility. MALDI relies on analytes being co-crystallized within a suitable laser energy absorbing matrix. Results of improper sample preparation, such as uneven matrix distribution, ion suppression, or the loss of low abundance compounds, can reduce analytical sensitivity. The dried droplet technique, which includes a mixture of matrix and analyte being pipetted and dried on a MALDI target plate, is the most used sample preparation technique as it allows homogenous co-crystallization to occur between the matrix and analyte prior to laser irradiation. The sublimation technique where the solid matrix is vaporized under a vacuum and is deposited as a dry, uniform film, is used in the high-resolution MALDI imaging of metabolites. MALDI also depends on reference spectral databases that contain mass spectral data, structural annotations, and biological context. For example, the Human Metabolome Database (HMDB) is the most comprehensive, curated database of human metabolites, containing over 220,000 metabolite entries, allowing for accurate mass matching and identification for cancer metabolomics studies. Quality guidelines for metabolomic studies and foundational reporting are provided by the Metabolomic Standards Initiative (MSI), established in 2007. It ensures that detailed documentation of sample preparation, storage, and collection is kept. As well as that, it categorizes metabolites detected by MALDI into one four confidence levels, describing the credence of their identification.

### 2.3. Improvements from 2000 to 2025

Between the years of 2000–2006, MALDI-MS moved from a proof of concept to actual applied tissue study. Sample preparation protocols like tissue sectioning, matrix deposition, and washing were described and standardized. These protocols allowed for obtaining metabolite signals from tissue rather than just proteins [21]. Since the mid-2000s, MALDI-MS TOF has highly improved pathogen identification in clinical labs thanks to its speed, accuracy, and cost-effectiveness. It allows species-level bacterial identification in labs to reach 84–94% success rates [22]. In the period of 2007–2012, MALDI-TOF instruments advanced and community reviews and method papers got together the best practices for imaging proteins, lipids, and small molecules. The number of atmospheric pressure MALDI variants and the adoption of clinical MALDI-TOF in microbiology also grew [23]. During 2013–2017, studies of matrix selection for lipids, matrix application methods such as sublimation and automated sprayers, and o-tissue derivatization methods were introduced. These new methods improved reproducibility and extended small-molecule coverage [24]. In 2015, MALDI-2 was developed and commercialized by Bruker Daltonics and featured two lasers; the first laser performed traditional MALDI, and a second laser re-ionized the neutral analytes that escaped ionization during the first laser. The implementation of two lasers had significantly increased ion yield often by 10–100×, which improved the coverage of metabolites and lipids in tissues. Between 2018–2021, MALDI-2, which addressed a major limitation of MALDI, that is, its relatively low ionization efficiency, had shown great sensitivity gains for small molecules like metabolites and glycans in multiple labs. MS/MS approaches and data processing pipelines for MSI developed. The standardization of matrix application and automated workflows increased output and in-lab reproducibility [25]. From 2022 to present day in 2025, MALDI-MSI studies have integrated MALDI-2, advanced matrices, high-resolution mass analyzers, and multimodal image registration. Demonstrations so far have shown large increases in metabolite coverage and tumor-specific metabolite discovery in prostate, RCC, colorectal, and other cancers [26].

### 2.4. Comparison with Other MS Techniques

MALDI-MS, in comparison with other MS techniques such as liquid chromatography mass spectrometry (LC-MS) and gas chromatography mass spectrometry (GC-MS), gives spatial maps of hundreds of molecules directly from tissue sections such as peptides, lipids, and many other metabolites, which is very useful for cancer heterogeneity and intra-tumor metabolic reprogramming. This is also excellent for connecting metabolite changes to histology and tumor micro-regions, biomarker discoveries, and spatial metabolomics in cancer-related studies [27]. MALDI-MS’s modern procedures can map many analytes at the same time from a single section and can be integrated with histopathology, which is best for lipids, many proteins, and medium- to high-*m*/*z* metabolites [28]. MALDI-MS still does have its limitations, such as the matrix application and matrix–ion suppression, making the detection and quantitation of some small polar metabolites difficult, and thus careful matrix choice and methods are needed. Researchers often follow up with LC-MS/MS for certain molecular IDs [29]. Other techniques, for example, LC-MS feature better chromatographic separation, higher sensitivity, a dynamic range, and more reliable identification, which makes it the workhorse for untargeted and targeted metabolomics [30]. GC-MS is also excellent for volatile and derivatizable small metabolites such as organic acids and amino acids after derivatization. MALDI-MSI is also commonly used in combination with LC-MS or GC-MS that identifies and quantifies spatially localized features. This combination of platforms gives the most powerful output in cancer studies [31]. But ultimately, MALDI-MS is preferred in cancer metabolite studies, as it gives excellent spatial maps of molecules.

## 3. Detection of Cancer Metabolites by MALDI from 2000–2025

### 3.1. Year 2000

**Figure 2:**
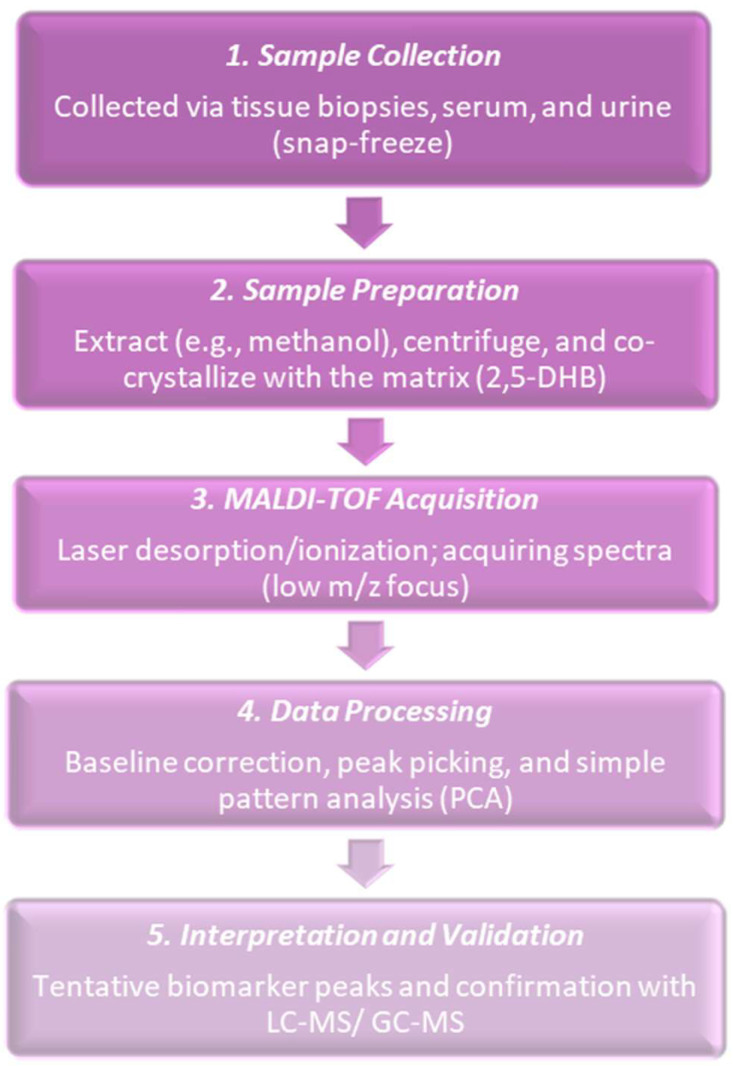
Five-step procedure followed in the MALDI-based detection of cancer metabolites during the year 2000. Early studies had pin-pointed its feasibility but also its limitations, such as sensitivity, mass accuracy, and reproducibility.

In 2000, researchers proved that it was possible to obtain biologically meaningful MALDI spectra from small, histological cancer populations and started applying MALDI imaging and profiling to cancer tissues. Sample preparation and matrix distribution issues that had been in practice were receiving attention and researchers worked on how to obtain reproducible tissue spectra. Their work emphasized matrix application, analyte distribution in matrix crystals, and imaging of matrix and analyte spots [32]. Commonly followed procedures at the time are shown in Figure 2. MALDI tissue imaging was being applied to the proteins and peptides of cancer, which showed the feasibility of molecular maps for the histology of tumors. MALDI-IMS researchers did early applications to breast cancer and other tumors, which showed that MALDI could map hundreds of molecular signals and connect them with histology. The direct acquisition of MALDI-TOF spectra from laser captured micro-dissected (LCM) transfer films was also demonstrated. Clear protein and peptide profiles were obtained from small cell populations (around 1250 cells) taken from human breast tissue. This made it possible to obtain molecular fingerprints from very small, histology-defined areas [33]. However, the problems in the routine detection of small polar metabolites in cancer tissues were not yet solved and only came after 2000 by means of new matrices, derivatization, and improved instruments [34]. At this time, MALDI in the world of cancer research was still mostly focused on proteins and peptides and not yet on broad small-metabolite profiling as that had needed more advances in matrices, derivatization, and instruments. Table 1 shows the notable research from the year 2000.

### 3.2. Year 2001

**Figure 3:**
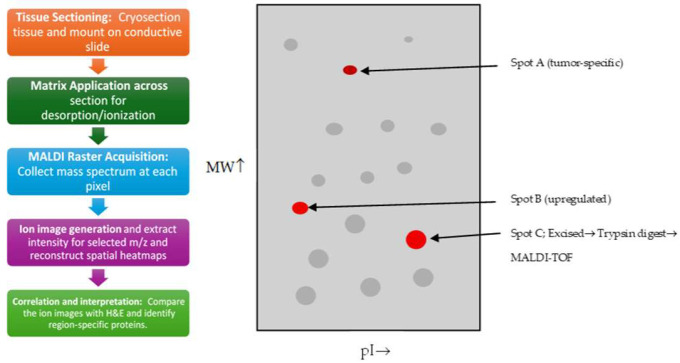
A simplified schematic of 2-D PAGE which involves loading a protein sample into a first-dimension gel to separate proteins by isoelectric point by Le Naour F et al., 2001 [42] that is showing protein separation by the isoelectric point (pI) and the molecular weight (MW). Grey spots are general proteins, while red spots are tumor-enriched proteins that are for excision and MALDI-TOF analysis. The letter A, B, and C are particular spots of three different protein.

MALDI imaging in 2001 shifted from a new technique to a validated, tissue-level tool for mapping proteins in tumor specimens. It had delivered well-accepted demonstrations such as the one by Stoeckli M et al. [36], as illustrated in Figure 3, and methodological work such as matrix and tissue handling and cross-platform comparisons, which set standards and led the next steps for metabolite detection and clinical application [37]. MALDI-TOF and MALDI-MS methods were being used in conjunction with LC/ESI for cancer cell and tissue proteome mapping, which solidified MALDI as an accessible tool for cancer metabolite detection [38]. Early evidence by several reports and studies in 2001 showed that MALDI was able to discriminate tumor margins and microenvironments, which was an important clinical application in surgical pathology and tumor characterization [39]. MALDI profiling was applied to compare normal tissue against tumor tissue, for example by profiling proteins from colon tumors in mouse models. This showed that MALDI could detect differential protein signatures that were associated with cancerous tissue, which further strengthened MALDI’s role in biomarker discovery in oncology [40]. Researchers and the authors were clear that MALDI-IMS excelled in imaging peptides, proteins, and many other lipids, but that small polar metabolite imaging and reliable molecular identification still required method improvements and complementary MS such as LC-MS or high-resolution MS, which ultimately resulted in the development of derivatization, better matrices, and high-resolution analyzers [41]. Table 2 shows the notable research from the year 2001.

### 3.3. Year 2002

**Figure 4:**
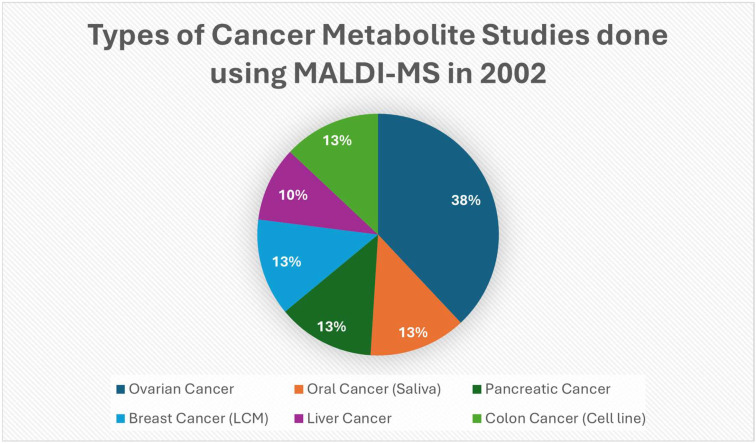
Pie shows the types of cancer represented in a selected set of 2002 PubMed studies that had clear examples of MALDI/MALDI-TOF/SELDI methods in 2002 to detect cancer-associated molecules. This is from a set of chosen examples with clear MALDI uses in 2002, not a detailed PubMed census [46,47,50,51,52,53].

By 2002, MALDI MS imaging and surface-enhanced laser desorption/ionization (SELDI) proteomic techniques had become acknowledged tools in cancer research, with more studies featuring clear examples of its use, as shown in [Figure 4]. SELDI is a variant of MALDI with a biochemical pre-enrichment step that is built directly into the target plate where different surface chemistries target specific properties of sample. For example, H4/H50 surface types will target C4-C18 alkyl chains and captures membrane-associated or hydrophobic proteins, and CM10 surface types will target carboxymethyl groups and captures basic plasma proteins and histones. The main aspect in 2002 remained on spatial protein and peptide mapping and biofluid profiling with improvements to sample prep and matrices methodology, which steadily improved sensitivity and paved the way for the metabolite-focused approaches that emerged soon after. Researchers were publishing more on matrix selection, deposition methods, and reproducibility in tissue-based MALDI analysis; this was important when targeting smaller molecules like lipids or metabolites [43]. SELDI introduced a way to enrich and detect low-abundance analytes in cancer, which led to bigger metabolite studies [44]. In 2002, MALDI, was beginning to get more recognition as a molecular histological tool due to its imaging power to map proteins and peptides directly on tissues. This maintained the spatial resolution, crucial for understanding tumor heterogeneity and biomarker detection [45]. Table 3 shows the notable research from the year 2002.

**Table 3:** Notable research from the year 2002.

No.	Author	Application	Result
1	Petricoin EF et al., 2002 [46]	SELDI serum proteomic profiling for ovarian cancer screening.	Identified a proteomic pattern in the serum that differentiated ovarian cancer patients from healthy patients.
2	Chen YC et al., 2002 [47]	Direct MALDI-TOF profiling of saliva from oral cancer patients and healthy patients.	Found altered saliva MALDI profiles with potential as a fast, non-invasive screening approach for oral cancer
3	Ball G et al., 2002 [48]	SELDI spectra and machine learning (ANN) to classify tumor types from proteomic patterns.	Showed that combining SELDI profiles with pattern-recognition could classify tumor sample.
4	Petricoin EF et al., 2002 [49]	SELDI serum profiling for prostate cancer detection.	Found spectral patterns that separated prostate cancer patients from non-cancerous patients, which helped popularize peptide-fingerprint approaches for cancer detection.

### 3.4. Year 2003

**Figure 5:**
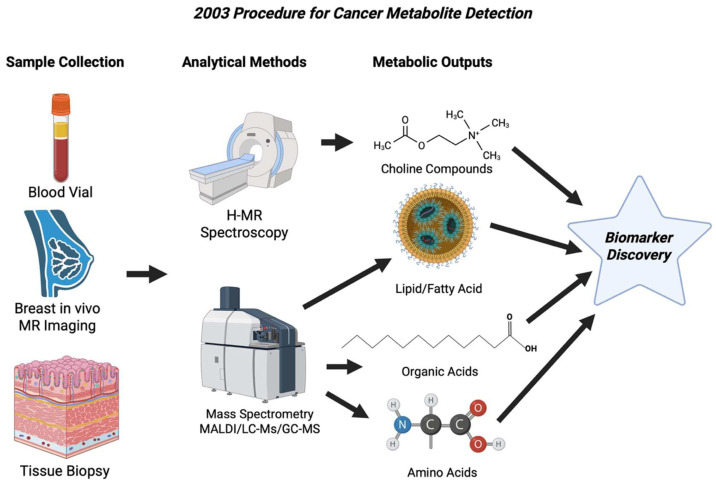
Workflow showing the main approaches to cancer metabolite detection in 2003. Clinical MR Spectrometry identified the choline peaks in breast tissue, while mass spectrometry identified lipids, organic acids, and amino acids in tissues [59].

MALDI developed from a proof of principle to a practical cancer application in 2003. A notable study showed proteomic patterns where MALDI spectra, acquired directly from small regions of frozen tissue, were able to classify non-small cell lung cancer subtypes, estimate nodal status, and separate patients by prognosis [54]. The best methods for tissue handling, sectioning, matrix choice, and deposition techniques were laid out, which is shown in Figure 5, that addressed important obstacles that had limited sensitivity and spatial fidelity for tumor analysis. The labs that had adopted these new methods saw better inter-sample reproducibility and more reliable metabolite detection [55]. Many studies in 2003 used SELDI to generate serum and proteomic signatures like hepatocellular carcinoma that was promising for screening or diagnosis. This increased the interest in MALDI-based metabolite detection but also triggered many questions on the study design, validation, and analytical reproducibility, which all ultimately led to better practice in the future [56]. Table 4 shows the notable research from the year 2003.

**Table 4:** Notable research from year 2003.

No.	Author	Application	Result
1	Yanagisawa K et al., 2003 [54]	MALDI tissue profiling of non-small cell lung cancer (NSCLC).	Showed proteomic patterns that classify NSCLC subsets and correlate with nodal status, which was evidence that MALDI can have strong clinical relevance.
2	Schwartz SA et al., 2003 [55]	Analyzed methods for tissue preparation, matrix deposition, and practical methods for tissue analysis.	Provided the best methods for tissue handling and matrix application that improved reproducibility and sensitivity for MALDI in cancer studies.
3	Reyzer ML et al., 2003 [57]	MALDI-QqTOF imaging of drug candidates or metabolites in tissue.	Showed that MALDI can map small-molecule drugs and metabolites in tissue and correlate with LC-MS, which meant MALDI is applicable across proteins and, now, small molecules.
4	Campa MJ et al., 2003 [58]	MALDI/Peptide profiling and follow up of identification of tumor associated proteins in lung cancer	Identified tumor associated proteins like MIF and cyclophilin A from tissue profiling showing useful biological targets from MALDI profiling.

### 3.5. Year 2004

**Figure 6:**
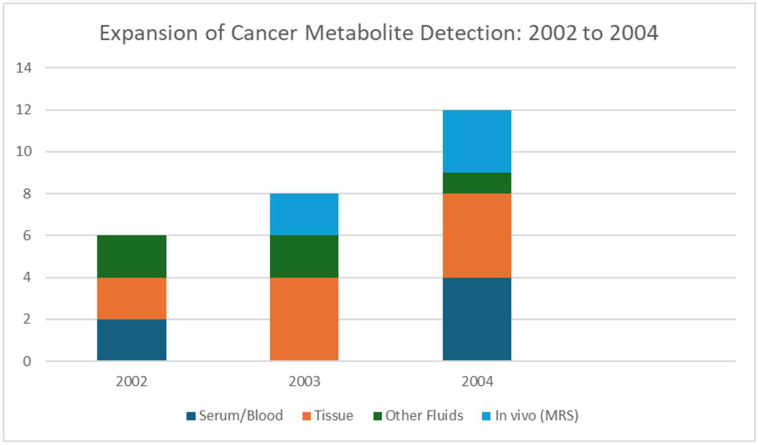
By 2004. Studies and analyses broadened in 2004 to include multiple cancer types, such as ovarian, prostate, breast, colorectal, and liver, and a wider range of sample sources, which included serum, tissue, and in vivo spectroscopy.

2004 was a transitional year for MALDI, as seen in Figure 6, that shows the increase in the types of cancers for which MALDI was used for the detection of metabolites. While MALDI in cancer metabolite imaging was not yet widespread, desorption/ionization on silicon (DIOS) gave new possibilities for matrix-free small-molecule detection and new methods for spatially mapping drugs with MALDI and gave way to metabolite MSI in cancer studies. MALDI-MS, with in situ digestion and imaging capabilities, was applied in 2004 to characterize tissue signatures after tumor vascular disruption therapy, while indirectly enabling metabolite-related insights [60]. In 2004, there was an emerging recognition of MSI’s potential across analytes. Reviews began articulating MALDI’s imaging capacity to visualize peptides, lipids, and small molecules in cancer tissues [61]. In general, 2004 did not feature many cancer metabolite MALDI studies, specifically the imaging of metabolites or lipids in tumors. Table 5 shows the notable research from the year 2004.

**Table 5:** Notable research from the year 2004.

No.	Author	Application	Result
1	Chaurand P et al., 2004 [62]	Coupling MALDI-IMS with histology in tumor tissues.	Showed practices relating MALDI molecular ion maps with stained histological images, thereby enabling region-specific molecular analysis, which was an important foundation for metabolite mapping.
2	Shen Z et al., 2004 [63]	Introduced DIOS for small-molecule detection.	Gave the proof of principle for matrix-free imaging of small molecules. It was a technical step towards metabolite imaging in cancer tissues.

### 3.6. Year 2005

**Figure 7:**
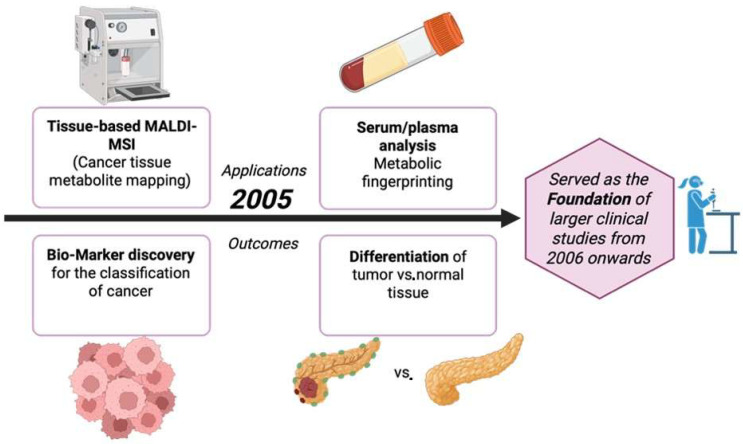
Applications and outcomes of MALDI-based cancer metabolite detection in 2005 [69].

The year 2005 introduced new methods in the world of cancer metabolite detection, which also led the way to the improvements made from 2006 onwards, as illustrated in Figure 7. MALDI profiling conducted directly on patient brain tumors showed that unlabeled mass spectra from tissue can differentiate prognosis. Although the results were mainly proteins, the clinical processes like fresh frozen tissue, histologically aligned spots, and statistics are all the platforms later used for on-tissue metabolites/lipids in cancer [64]. Three-dimensional registration and visualization of MALDI images was introduced and metabolite and lipid maps were set up to be compared against tumor microanatomy, like its necrosis and vasculature [65]. A huge breakthrough in spatial resolution, around 25 µm, was achieved, which is crucial in mapping small metabolites and lipids inside heterogenous tumor regions [66]. Reviews also laid out matrices, coating, sectioning, and data-analysis methods for on-tissue small metabolites. These methods were later also applied to tumor metabolites and lipids [62]. Table 6 shows the notable research from the year 2005. 

**Table 6:** Notable research from year 2005.

No.	Author	Result	Application
1	Schwartz SA et al., 2005 [64]	Direct tissue MALDI profiling of human gliomas in clinical pathology.	Showed MALDI-based tissue study could classify gliomas and help prognosis. This led the path for MALDI clinical classifiers.
2	Jurchen JC et al., 2005 [66]	MALDI-MSI oversampling to boost laser-spot limits.	Found how to show features smaller than the laser beam, thereby boosting spatial resolution. This was important for mapping heterogenous tumor metabolite environments.
3	Rohner TC et al., 2005 [67]	Methods for MALDI-MSI of biological tissue sections.	Explanation of MALDI-MSI for tissues, which became the cornerstone for later metabolite and small-molecule cancer imaging.
4	Jackson SN et al., 2005 [68]	In situ characterization of phosphatidylcholines in tissue by MALDI-TOF.	Found on-tissue structural identification of lipid metabolites, which became a key feature applied to cancer lipidomics by MALDI-MSI.

### 3.7. Year 2006

**Figure 8:**
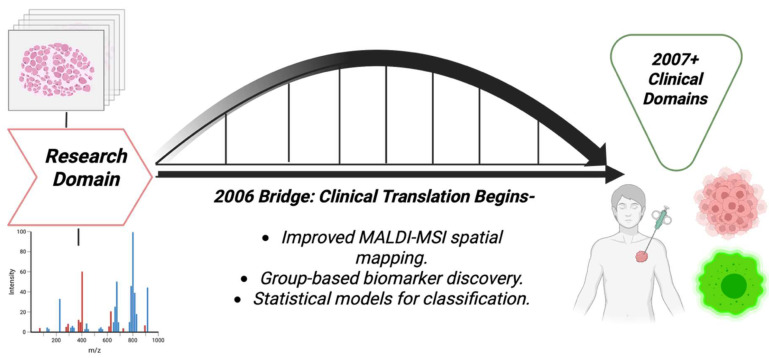
Bridge pathway diagram that shows the transitional nature of 2006, moving from mainly research domains to clinical domains later.

Several technical advancements were made in 2006, and removed major barriers to the sensitive, spatial detection of small molecules and lipids in tissue. Whole-body MALDI imaging of animal tissue sections was demonstrated, proving that MALDI-IMS can map many endogenous and exogenous species in large tissue areas. This was a huge technical step toward spatial metabolite detection in disease models and cancer pharmacology [70], as shown in the [Figure 8] bridge illustration. A histological way of tissue profiling was introduced, that is, the robotic deposition of matrix onto regions selected by pathologists for breast cancer, thereby improving cellular specificity and efficiency. That way of histological profiling was technology that allowed small-molecule or lipid signals to be linked to specific histological features like tumors, stroma, and margins, which was important for cancer metabolite discoveries [71]. MALDI serum and biofluid profiling still remained in use for cancer detection, for example in colorectal cancer profiling, which, although was assuring the potential of MS-based pattern detection, also raised concerns about reproducibility and biomarker validation [72]. Table 7 shows the notable research from the year 2006.

**Table 7:** Notable research from year 2006.

No.	Author	Application	Result
1	Cornett DS et al., 2006 [71]	Histological MALDI profiling in breast cancer, particularly robotic matrix deposition on regions selected by pathologists.	Showed a process that increased cellular specificity and processing for tissue profiling which enabled region-specific molecular comparisons between tumor and stroma.
2	de Noo ME et al., 2006 [72]	MALDI-TOF serum protein profiling for colorectal cancer detection.	Large serum study showing high sensitivity and specificity around 95% for CR C classification using MALDI serum profiles. This strengthened serum profiling’s potential and the need for validation.
3	Aerni HR et al., 2006 [73]	Automated acoustic droplet matrix deposition for MALDI sample preparation.	Introduced high-processing, reproducible acoustic matrix deposition, which improved spot uniformity and sensitivity. This reduced analyte delocalization and improved the detection of low-abundance species.
4	Sampson JS et al., 2006 [74]	Hybrid MALDI-ESI demonstration for the generation and detection of multiply charged peptides and proteins by laser desorption electrospray ionization.	Found MALD-ESI as an alternate ionization method, boosting small molecules and multiply charged species detection, which provided more options for metabolite ionization and improved MS/MS performance.

### 3.8. Year 2007

**Figure 9:**
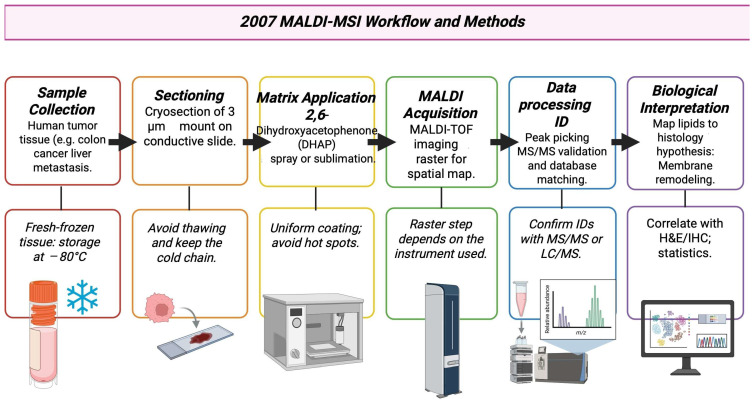
Example method pathway based on 2007 MALDI-MSI literature by Shimma S et al., 2007 [75,80].

MALDI imaging moved from a proteomics-focused research tool to a usable, practical method in lipid and small molecule mapping in cancer tissue. Primary reports from 2007 show that MALDI-MSI could map phospholipids and other small molecules in cancer specimens, for example, colon cancer and liver metastasis showed subnormal local lipid distributions that relate to tumor regions [75]. Research groups used more on-tissue MS/MS, tandem TOF/TOF, or high-resolution analyzers to comment on lipid or metabolite ions observed by imaging. This lowered the uncertainty in assigning tumor-localized signals to specific metabolite species [76]. The workflow commonly followed in 2007 is shown in Figure 9. Work conducted in 2007 also identified matrices and matrix application methods such as dihydroxyacetone phosphate (DHAP)/dihydroxyacetone (DHA) and optimized deposition, both of which improved the detection of hydrophobic lipids and other low-mass species, which in turn improved cancer metabolite imaging [77]. Table 8 shows the notable research from the year 2007.

**Table 8:** Notable research from year 2007.

No.	Author	Application	Result
1	Shimma S et al., 2007 [75]	MALDI-IMS mapping of phospholipids in colon cancer and liver metastasis.	Showed abnormal, region-specific distributions of phospholipid species in colon cancer metastasis and used MS/MS to support molecular identification. This was early proof that MALDI-IMS can map endogenous lipids in cancer tissue.
2	McDonnell LA et al., 2007 [76]	Detailed review of imaging mass spectrometry, covering analyte classes and instrumentation.	Compared ionization methods, matrices, spatial resolution and data analysis. This gave the best practices for lipid and metabolite imaging and became adopted in cancer studies.
3	Schwamborn K et al., 2007 [78]	Tissue profiling and classification in prostate cancer by MALDI imaging.	A clinical study with 22 prostate sections showed MALDI-IMS protein expression patterns that separate cancerous and non-cancerous regions. It was evidence of its clinical uses for tumor molecular mapping.
4	Altelaar AFM et al., 2007 [79]	A practical guide for MALDI imaging at cellular length scales.	Gave guidelines for sample preparation, matrix deposition, and measurement at near-cellular resolution; this made high quality, spatially resolved lipid and metabolite measurements in tumors more reproducible.

### 3.9. Year 2008

**Figure 10:**
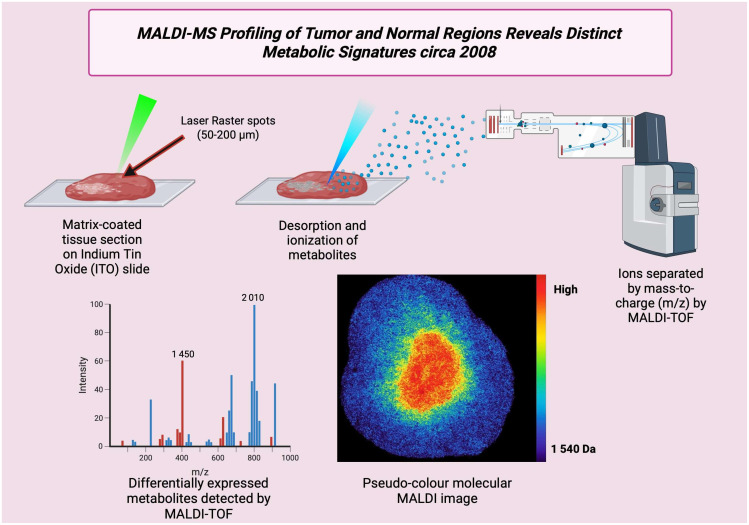
Schematic of MALDI profiling of a cancer tissue section (circa 2008). MALDI enables spatially resolved analysis of metabolites and small proteins directly from tissue sections.

In 2008, major advances made MALDI imaging more applicable to actual clinical samples, like FFPE and alternative preservation. It also improved mass-resolution approaches for small molecules and drug metabolites and stronger statistics [Figure 10] and image analysis processes to understand complex tissue maps. High-mass-resolution FT-ICR MALDI showed that drugs and small metabolites could be viewed with high mass accuracy, which improved confidence in metabolite assignments from tissues [81]. Alternate preservation methods like on-tissue proteolysis, alcohol preservation, and improved sample preparation were found, thereby opening access to large clinical tissue archives for MS research. This increased MALDI’s clinical translation potential [82]. Applied cases showed MALDI mapping of drug bioavailability and metabolite localization in tumor xenografts, which showed direct translational use for cancer therapy studies, for example, AEE788 distribution in prostate xenografts [83]. Many publications introduced hierarchical clustering and other multi-variety approaches for MALDI images of complex cancer, thereby improving objectivity and lesion classification [84]. Table 9 shows the notable research from the year 2008.

**Table 9:** Notable research from year 2008.

No.	Author	Application	Result
1	Groseclose MR et al., 2008 [82]	On-tissue tryptic digestion and MALDI-IMS applied to FFPE tissue (lung tumors).	Found reproducible peptide and protein imaging across TMA cores and related it with histology. This opened the use of archived clinical FFPE cancer samples for MALDI studies.
2	Huamani J et al., 2008 [83]	Prostate tumor study that used MALDI to examine EGFR/VEGFR inhibitor AEE788 in xenografts.	Found intratumoral drug distribution (AEE788) related with different treatment responses between models; it was a clear case where MALDI detected a therapeutic and its spatial distribution in cancer tissue.
3	Deininger SO et al., 2008 [84]	Applied hierarchical analysis to MALDI imaging datasets from cancers to segment tissue regions.	Introduced approaches that separate MALDI images into tumor vs. non-tumor regions. This improved analysis of metabolite detection processes.
4	Chaurand P et al., 2008 [85]	Tested alcohol-preserved tissue (EPEE) as a replacement for FFPE for intact protein MALDI imaging.	Showed that alcohol preservation allows the imaging of intact proteins, thus avoiding formalin crosslinking problems. This gave a new way to analyze preserved cancer tissue.

### 3.10. Year 2009

**Figure 11:**
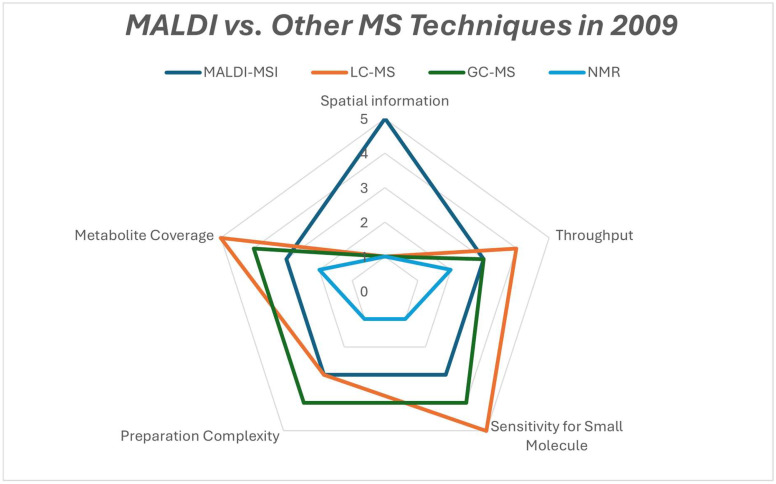
Radar comparison chart of the strengths of metabolomics platforms that were used in 2009.

The year 2009 saw MALDI tissue imaging and serum and plasma profiling shift towards clinically applicable cancer studies, with many researchers considering a diagnostic model for colon cancer and other cancers [86]. MALDI-TOF and FT-ICR profiling studies had been conducted to create spectral libraries in NIST style for colorectal and other cancers, which improved case comparability and diagnosis efforts [87]. MALDI-FT-ICR and other associated methods [Figure 11] for detecting glycan biomarkers in breast, ovarian, and prostate cancer serum were developed and validated in 2009 [88]. MALDI was able to identify tissue-localized protein fragments with diagnostic powers, for example, a MEKK2 fragment differentiating prostate cancer and non-cancer tissue. This showed its ability to detect metabolites [89]. Many studies featured sample preparation and fractionation methods to reduce complexity and data processing, like in various classification models. This increased reproducibility in tumor versus normal classification, particularly in lung cancer subtypes [90]. Table 10 shows the notable research from the year 2009.

**Table 10:** Notable research from year 2009.

No.	Author	Application	Result
1	Cristoni S et al., 2009 [87]	Testing a MALDI spectral reference in a NIST-style method for colorectal cancer tissue spectra to improve sample classification.	Showed that comparing patient MALDI spectra with a selected spectral library improved the classification of cancerous vs. normal colorectal tissue. This was a step toward standardized identification of metabolites when present in spectra.
2	Barkauskas DA et al., 2009 [88]	MALDI-FT-ICR profiling of released serum glycans from breast, ovarian, and prostate cancer	Developed statistical methods for high-resolution MALDI-FT-ICR glycan data and found glycan features that differentiate cancer and normal tissue, thus showing MALDI’s use in glycan metabolite detection in cancer
3	Cazares LH et al., 2009 [89]	MALDI-MS imaging of prostate tissue to find molecular signals that differentiate tumor and non-tumor regions.	Identified an *m*/*z* 4355 fragment (MEKK2) that differentiated prostate cancer from non-cancerous tissue. This was an important study that put forth processes used for metabolite imaging.
4	Acquadro E et al., 2009 [91]	MALDI-IMS detection of a MRI contrast agent in mouse tissue to show the detection of small molecules by MALDI.	Found that MALDI-IMS can detect, map, and identify a small molecule contrast agent; these methods were directly applied to the imaging of metabolites in tumor tissue.

### 3.11. Year 2010

**Figure 12:**
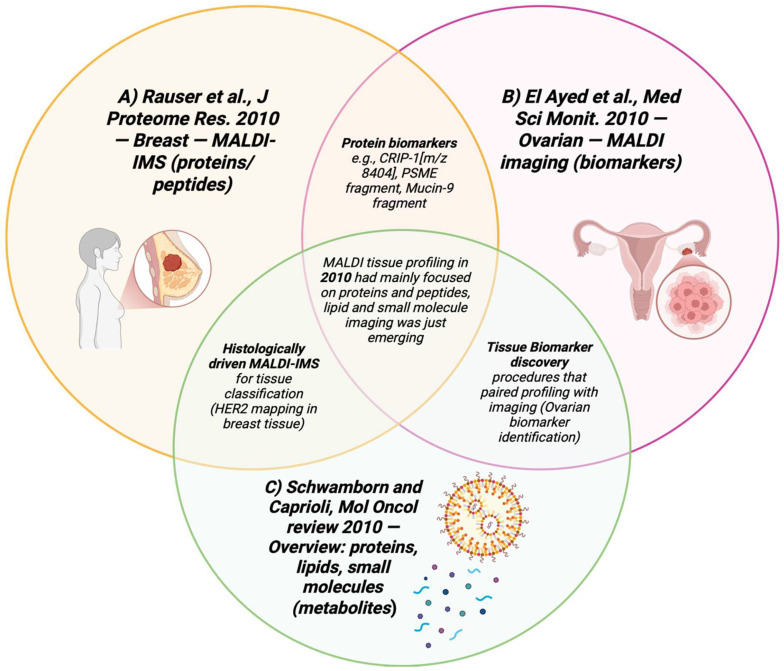
Venn diagram summarizing the applications of 2010 articles that used MALDI-MSI in breast and ovarian cancer tissues [95,96,99,100].

2010 was characterized by many innovations in methods and early demonstrations of metabolite imaging. Although MALDI was still mainly centered on proteins and peptides, several studies began to state the use of MALDI in small molecule cancer metabolite detection. Lipid-based MALDI imaging was applied to frozen non-small cell lung cancer (NSCLC) and nearby normal tissue, where phosphatidylcholine and sulfatide species were found to differentiate between tumor types accurately [92]. Innovations like matrix nanoparticles (fNP), new solvent procedures, and organic salt pre-treatments allowed for the better visualization of lipids and small metabolites at high spatial resolution ~15 µm [93]. The methods for combining glycolic profiling with MALDI-MSI were emerging, and due to this, the spatial detection of tissue glycans became more feasible in the years that followed [94]. MALDI-TOF imaging of meningioma progression showed how mass imaging of methods could be altered to capture metabolic shifts in tumor evolution, once again framing the groundwork for metabolite detection [45]. Table 11 shows the notable research from the year 2010. Figure 12 summarizes key MALDI studies conducted in 2010.

**Table 11:** Notable research from year 2010.

**No.**	**Author**	**Application**	**Result**
1	Rauser S et al., 2010 [95]	MALDI-IMS was applied to freshly frozen breast cancer sections to classify HER2 using spatially resolved molecular profiles.	Showed that MALDI-IMS can HER2 status with high accuracy, sensitivity ~83%, specificity ~92%. The processes used are the same ones used for metabolite imaging in caner tissues.
2	Schwamborn K, Caprioli RM, 2010 [96]	Review on MALDI methods and application in oncology, including the detection of cancer metabolites.	Found that MALDI can map glycans and small metabolites in tissues and MS settings that allow metabolite imaging, thus showing MALDI as a great tool for spatial metabolomics in cancer research.
3	Colsch B, Woods AS, 2010 [97]	Used MALDI to detect sialylated glycosphingolipids in tissue sections using optimized matrices.	Showed spatially resolved detection of ganglioside and glycosphingolipids of *m*/*z* < 950 from tissue without derivatization. While conducted on brain tissue, these methods are translatable to cancer studies where and glycosphingolipid metabolism is important.
4	Iorio E et al., 2010 [98]	An ovarian cancer study that altered phosphatidylcholine metabolism in cancer cells.	Found the activation of phosphatidylcholine cycle in ovarian cancer cells and gave biochemical targets and mass signatures that MALDI later imaged in tumor tissue. It showed important context on which metabolites were oncologic for MALDI detection.

### 3.12. Year 2011

**Figure 13:**
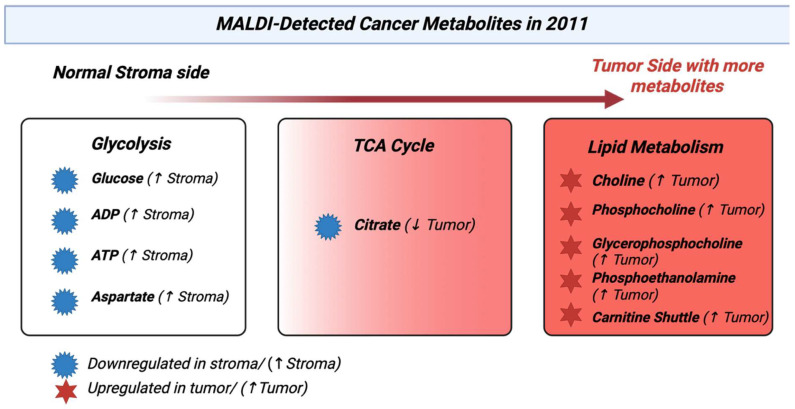
Heatmap-pathway showing the metabolic changes reported by MALDI in tumors [107].

By 2011, MALDI had moved from just proteins to lipid and metabolite signatures in tissue that were being used to classify cancers. MALDI profiling of lipids could differentiate tumors from nearby normal tissue, as seen in Figure 13, and classify subtypes, for example, intrinsic breast cancer subtypes from surgical sections. This showed MALDI’s growing role in metabolite tumor detection [101]. MALDI lipid profiles in intrahepatic cholangiocarcinoma showed phospholipid alterations that differentiated cancer tissue. It was an early showing of lipid metabolite signatures in hepatobiliary malignancy [102]. An important sequential DESI and MALDI imaging process had enabled lipid maps through DESI and protein maps through MALDI from the same tissue section while preserving the morphology. This was an approach that was widespread later to connect metabolite and proteomics methods in cancer studies [103]. Many reviews from 2011 noted that MALDI could be used to measure metabolites and lipids directly from tissue, which supported the detection of cancer metabolites. [104]. Table 12 shows the notable research from the year 2011.

**Table 12:** Notable research from year 2011.

No.	Author	Application	Result
1	Kang HS et al., 2011 [101]	Breast cancer tissue lipidomics by MALDI imaging.	Certain phospholipids like PI, PE, PC showed tumor-related spatial patterns in breast cancer, supporting lipids as diagnostic markers.
2	Park YS et al., 2011 [102]	Cholangiocarcinoma vs. normal liver by MALDI imaging of phospholipids.	Multiple phospholipids were differentiated in tumors vs. nearby liver tissue, thus enabling the differentiation of cholangiocarcinoma.
3	Wang J et al., 2011 [105]	Colorectal cancer metastasis (through mouse xenograft) metabolite MALDI-TOF imaging.	On tissue, MALDI-IMS mapped small metabolites and lipids like UDP-HexNAc, glutathione, and phospholipids differing between tumor and stroma, showing metabolic reprogramming in metastases.
4	Han EC et al., 2011 [106]	Hepatocellular carcinoma direct tissue MALDI analysis, particularly in the low mass protein regions.	Showed MALDI tissue profiling to separate tumor and non-tumor regions. It set the basis for molecular mapping in HCC that proliferated in lipid-focused studies.

### 3.13. Year 2012

**Figure 14:**
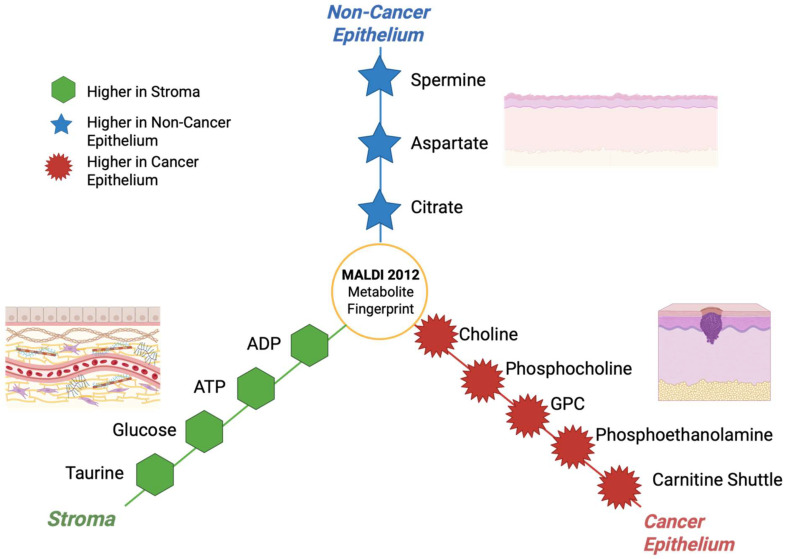
Hub and spoke illustration showing the compartment-specific metabolites fingerprints that were detected by MALDI-MSI in 2012 [113].

Multiple groups in 2012 used MALDI on human tumor tissues to classify cancers and map tumor versus non-tumor regions using lipid signatures, especially in non-small-cell lung cancer (NSCLC). These studies built a base of practical sample preparation and analysis, which is still being developed today [92]. The year 2012 also had many important method papers showing how to extend MALDI to low-mass metabolites [Figure 14] like energy and central-carbon metabolites using 9-aminoacridine (9-AA) and optimized negative ion conditions. This was a step that was later conducted on cancerous tissue [108]. Although most glycan imaging studies were published between 2013–2014, the concept and processes that would allow MALDI to profile N-linked glycans directly in tissue including FFPE blocks were coming together around 2012 and after, which set up many cancer applications [109]. Table 13 shows the notable research from the year 2012.

**Table 13:** Notable research from year 2012.

No.	Author	Application	Result
1	Lee GK et al., 2012 [92]	NSCLC classification from bronchoscopic diagnosis using MALDI lipid profiling.	Lipid profiles accurately differentiated tumors from nearby normal tissue and classified NSCLC histologic type with an 80% accuracy.
2	Cerruti CD et al., 2012 [110]	9-aminoacridine (9-AA) MALDI imaging for low-mass/negative-ion detection.	Showed that 9-AA matrix with optimized negative-ion MALDI gives clean, low-mass metabolite and lipid spectra, which became an advanced method commonly used for imaging polar metabolites like lactate and succinate in cancer tissues.
3	Yang HJ et al., 2012 [111]	MALDI 15-T FT-ICR analysis of cancer cell lipids.	Identified intact phospholipids from malignant glioma cells by MALDI with high resolution and high mass accuracy. This improved reliability in metabolite assignments in cancer studies.
4	Maurer K et al., 2012 [112]	MALDI-TOF profiling of oral brush biopsies for the early detection of cancer	Found a clinical application showing MALDI profiling of invasive oral samples that can discriminate malignant and premalignant cancer from benign regions.

### 3.14. Year 2013

**Figure 15:**
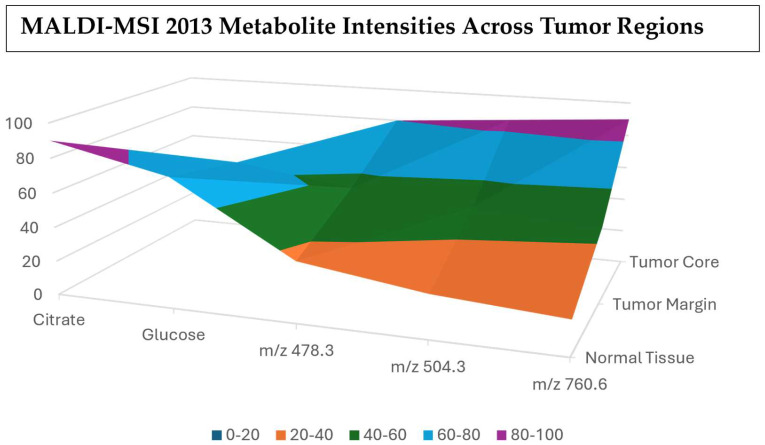
Metabolite intensity waterfall chart showing the stepwise changes across the different tissue regions [119].

In 2013, cancer metabolite detection by MALDI had moved from just studies to actual large-scale tissue microarrays (TMAs) with hundreds of patient samples. This showed that MALDI-MSI could generate reproducible metabolite signatures even in other large research groups, thus making it more clinically relevant [114]. MALDI procedures were applied to routine clinical specimens like formalin-fixed paraffin-embedded tissues, biopsies, and resection samples, which further showed the feasibility of integrating MALDI in hospital pathology procedures for cancer diagnosis and prognosis [115]. Even though peptides and proteins were still the main focus, more and more researchers had started broad studies on lipid signatures, amino acid derivatives, and small metabolites detectable by MALDI. Figure 15 shows the intensities of metabolites across different types of tissues. These studies found tumor-associated metabolic reprogramming, for example, the changes in membrane liquid composition and oxidative stress-related metabolites [116]. Table 14 shows the notable research from the year 2013.

**Table 14:** Notable research from year 2013.

No.	Author	Application	Result
1	Pirman DA et al., 2013 [116]	Direct MALDI-MS analysis of colorectal cancer cells to detect metabolic changes without the prior separation step.	Found cancer-specific metabolic reprogramming, altered energy metabolism, and signaling pathways, thereby showing MALDI’s ability to track functional metabolic shifts in tumor cells.
2	Quaas A et al., 2013 [117]	MALDI imaging of esophageal carcinoma to study metabolite features in adenocarcinoma and squamous cell carcinoma.	Showed tumor-associated peptide signatures differentiating adenocarcinoma from squamous cell carcinoma. This showed MALDI’s potential in subtyping and precision diagnosis.
3	Pallua JD et al., 2013 [118]	Used MALDI-MSI to examine prostate cancer tissues with a focus on metabolite detection for clinical use.	Detected overexpression of biliverdin reductase-B, which is a redox enzyme in prostate cancer samples, thus taking it as a new biomarker type with implications for oxidative stress regulation in tumors.
4	Alexandrov T et al., 2013 [119]	Developed computational segmentation algorithms for MALDI imaging datasets from larynx carcinoma tissue.	Achieved better differentiation of tumor and normal tissue regions, thereby giving a better understanding of complex imaging data.

### 3.15. Year 2014

**Figure 16:**
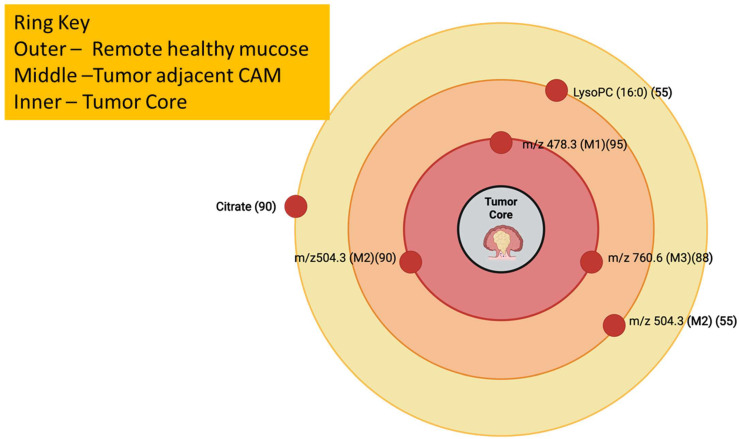
Cancer-Adjacent Metaplasia (CAM) Concentric-Zone Map with lipid signatures mapped across remote, CAM, and tumor, which reflects Mirnezami et al., 2014. The numbers beside the *m*/*z* values are in parentheses [120,127].

A MALDI study on fresh frozen tissue sections obtained from colorectal cancer patients traced clear phospholipid signatures in tumor, adjacent, and distant tissue regions. This showed cancer-adjacent metaplasia (CAM) [Figure 16], which are metabolic changes in healthy mucosa near tumors [120]. Ambient ionization mass spectrometry was used intraoperatively to trace metabolites during brain tumor surgeries. This allowed real-time metabolic detection to guide resection, a huge leap toward the clinical translation of metabolite-guided cancer study [121]. Reviews in 2014 also mentioned the ability of MALDI to image endogenous metabolites and dietary phytochemicals in tissues. It showed the advances in matrix selection, metabolite coverage, and challenges related to the methods being used in cancer metabolite detection [122]. The tumor microenvironment in breast cancer was studied by many different centers, and the proteomic differences in cancer-associated stromal regions were identified by across these centers. This showed scalable MSI applications that were relevant to metabolite tracing in tumors [123]. Table 15 shows the notable research from the year 2014.

**Table 15:** Notable research from year 2014.

No.	Author	Application	Result
1	Mirnezami R et al., 2014 [120]	MALDI imaging performed on colorectal cancer resections to chemically trace tumor, surrounding mucosa, and stroma, putting focus on small metabolite signatures.	Discovered CRC-related metabolic “field effects” in normal mucosa with modified phospholipid and sphingolipid patterns and other multivariate models were able to differentiate microenvironments.
2	Jones EE et al., 2014 [124]	MALDI imaging on clear cell renal cell carcinoma (ccRCC) tissue trace low-mass metabolites with protein signals and clinical pathology.	Found panels of tumor-related lipids around 39 species and proteins around 26 that could separate tumor and non-tumor tissue and ranked its recurrence risk. This was proof that spatial lipid metabolism helps ccRCC phenotype.
3	Toue S et al., 2014 [125]	Found on-tissue chemical derivatization (THAS) to allow the high-contrast imaging of endogenous amino acids, that are key cancer metabolites, on tissue sections.	Was able to visualize more than 20 amino acids with cellular scale contrast, thereby finding a robust process to trace glycolysis and TCA-related metabolites in tumors by MALDI.
4	Guo S et al., 2014 [126]	MALDI-FT-ICR imaging of resected thyroid tumors along with serum lipidomics to find metabolic biomarkers.	Were able to find region-specific tumor lipid alterations in thyroid tissues and serum lipid specimens supporting the differentiation of tumors and benign- joint tissue–serum metabolite process.

### 3.16. Year 2015

**Figure 17:**
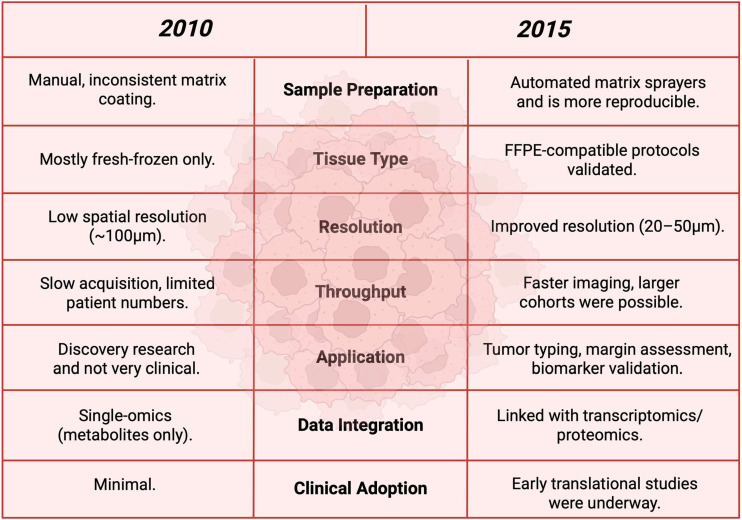
A before-and-after comparison table between the year 2010 and 2015 to show the advancements in between these years [132].

In 2015, specialized matrices allowed for true ‘metabolite level’ imaging in tumors. N-(1-naphthyl) ethylenediamine dihydrochloride (NEDC) made MALDI able to image very small metabolites, including glucose, in colorectal cancer and liver metastases, which showed intra-tumoral glucose gradients in accordance with the Warburg Effect. This was a technical step in increased sensitivity and negative-ion performance for low *m*/*z* metabolites [105]. The advancements in the five years in between 2010 and 2025 are shown in Figure 17. A clinical–pathological process showed that high-resolution MALDI-FT-ICR on formalin-fixed paraffin-embedded (FFPE) tissues could trace small metabolites and relate them to histology and thus, had opened large, archived biobanks to be used with spatial metabolomics in metabolite detection [128]. MALDI was able to trace hypoxia-driven changes in breast tumors across different metabolic pathways like glycolysis and membrane remodeling. From this, it was able to connect oxygenation status to spatial molecular phenotypes and understand how microenvironment signals can affect tumor metabolism [129]. Table 16 shows the notable research from the year 2015.

**Table 16:** Notable research from year 2015.

No.	Author	Application	Result
1	Buck A et al., 2015 [128]	MALDI-FT-ICR performed on FFPE cancer tissues to test if endogenous metabolites can survive and be imaged.	Showed in situ imaging of metabolites from FFPE with high mass resolution and big overlap to fresh frozen spectra, with which it opens archival clinical cohorts to metabolite MSI.
2	Jiang L et al., 2015 [129]	3D MALDI MSI of breast tumor xenografts that were engineered with a hypoxia reporter; integrated lipid and tryptic peptide maps to study hypoxia-driven metabolic remodeling.	Were able to identify co-localization of hypoxic regions with distinct phospholipids and hypoxia-regulated pathways, showing how hypoxia shapes tumor lipid metabolism spatially.
3	Goto T et al., 2015 [130]	High-resolution MALDI-IMS of phospholipids in human prostate cancer resection specimens relating lipid distribution to clinical outcomes.	Found decreased LPC (16:0/OH) in cancer regions; this reduction in lipid single-handedly predicted biochemical recurrence, thus a lipid-metabolism biomarker of aggressiveness.
4	Kriegsmann J et al., 2015 [131]	MALDI-TOF imaging was applied in clinical pathology (FFPE tissue analysis).	MALDI-TOF MSI detected small molecules like lipids, carbohydrates, and small peptides in FFPE and discussed about its potential for biomarker discovery and delineation of tumor margins.

### 3.17. Year 2016

**Figure 18:**
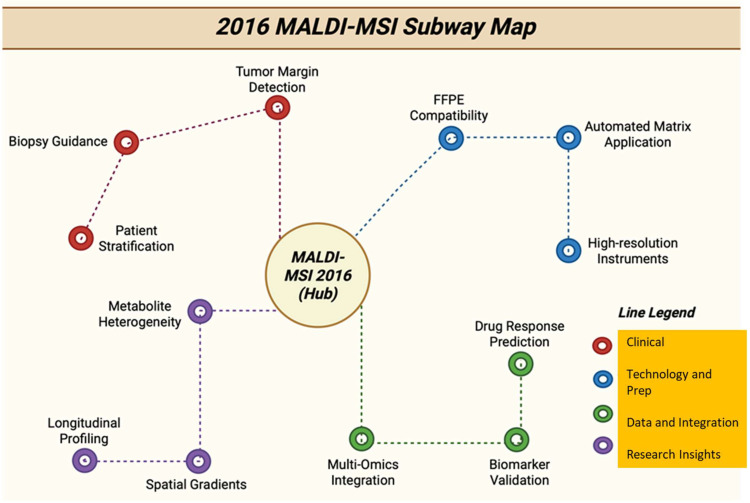
Subway map with tracks showing 2016 MALDI-MSI directions, from methods and instruments to clinical applications [139].

In 2016, the research community showed keen interest in the way that MSI results are presented, validated, and quantified, for example, the standardization of visualization, QA/QC, and statistics. This increased the confidence in metabolite assignments and in the translation of spatial metabolomics to clinical research [133]. [Figure 18] shows the various directions of MALDI in 2016. Processes started appearing that reliably profile N-linked glycans from (FFPE) tumor sections, allowing retrospective studies on archived clinical cohorts and related glycosylation patterns to tumor regions [94]. Multiple studies used MALDI imaging to localize chemotherapeutics (e.g., tamoxifen, irinotecan/payloads, paclitaxel) and their metabolites inside tumor tissue or 3D cell models, showing intratumoral heterogeneity in drug penetration and local metabolism, which is important for pharmacology and metabolite-driven resistance. MALDI studies in 2016 also reported the molecular discriminators of metastasis and lymph-node involvement, which are protein and metabolite ions, supporting these metabolite-based signatures as markers of metastatic potential [134]. Table 17 shows the notable research from the year 2016.

**Table 17:** Notable research from year 2016.

No.	Author	Application	Result
1	Végvári Á et al., 2016 [135]	MALDI-MSI localization of tamoxifen and metabolites in human breast tumor sections.	Explained the intratumoral distribution of tamoxifen and its metabolites, showing that MALDI can trace small molecules and metabolites in clinical tumor tissue and let us know metabolism relationships.
2	LaBonia GJ et al., 2016 [136]	MALDI imaging of irinotecan penetration and metabolism in 3D tumor spheroids treated in a 3D printed fluidic device.	Showed the spatial penetration and metabolic conversion of irinotecan within spheroids including the localization of active metabolites, displaying MALDI’s ability to study intratumoral drug metabolism and microenvironmental barriers.
3	Giordano S et al., 2016 [137]	MALDI -MSI of paclitaxel distribution on various tumor models like ovarian, colon, breast, and mesothelioma.	Found marked heterogeneity in paclitaxel distribution between tumor types, showing that the metabolite spatial mapping explains the variable therapeutic outcomes.
4	Patterson NH et al., 2016 [138]	Lipid MSI of colorectal cancer with liver metastases to have a pathological response to therapy.	Produced lipid signatures related with pathological response to neoadjuvant chemotherapy, thus showing that lipid metabolite maps can serve as objective markers of the effect of treatment.

### 3.18. Year 2017

**Figure 19:**
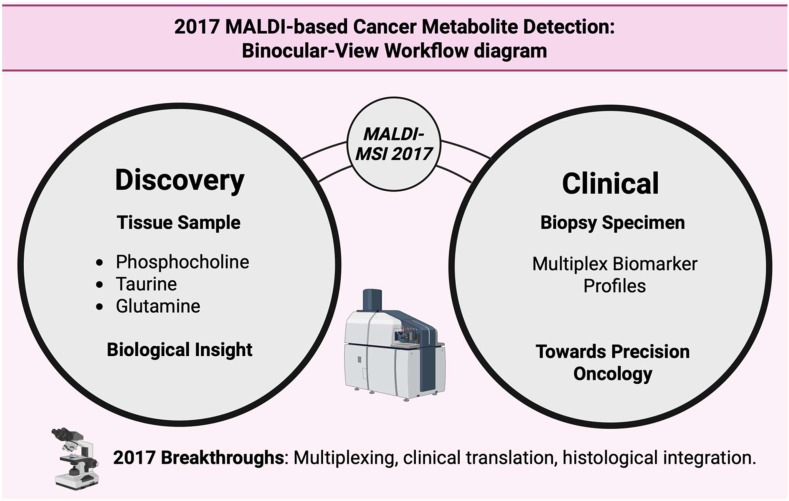
The dual-lens perspective integrates metabolite discovery with clinical biomarker profiling to advance precision oncology [145].

By using MALDI-FT-ICR MSI with matrix coating techniques (MCAEF) and multiple matrices like quercetin and 9-aminoacridine, researchers were able to detect and localize over 1000 endogenous metabolites in prostate tissue, showing the metabolism dysregulation between cancerous and non-cancerous regions [12]. High-field FT-ICR MALDI, for example 15-T, enabled the resolution of isobaric and isotopic overlaps that had confused metabolite signals and allowed the confident alignment of on-tissue MSI signals with LC-MS micro proteomics from micro dissected regions. This improved the molecular specificity for metabolites and small molecules in tumor sections [140]. Handheld ambient sampling methods like MasSpec Pen were introduced in 2017 and showed fast, nondestructive sampling that produced metabolic fingerprints for real-time classification. The MasSpec Pen showed remarkable sensitivity and specificity in in vivo mice experiments and many different human samples [141]. Table 18 shows the notable research from the year 2017. Figure 19 shows how MALDI was used in both metabolite discovery and clinical profiling.

**Table 18:** Notable research from year 2017.

No.	Author	Application	Result
1	Zhang J et al., 2017 [141]	Developed the MasSpec Pen which is a handheld, nondestructive ambient MS sampling method, for fast ex vivo and in vivo tissue analysis during surgery.	Gave close to real-time metabolic fingerprints that differentiated tumor vs. normal tissue on many human sample types; this was a huge step towards intraoperative metabolite-based diagnostics.
2	Dilillo M et al., 2017 [142]	Applied ultra-high mass resolution 15-T MALDI FT-ICR on a mouse glioblastoma model to trace proteins, lipids, and small metabolites.	Resolved isobaric and isotopic overlaps and traced hundreds of proteins and metabolites with high confidence, thus improving molecular specificity for on-tissue metabolite work.
3	Casadonte R et al., 2017 [143]	Review on MALDI applied to cancer tissue microarrays (TMAs) on clinical groups.	Showed how MALDI processes scale to large clinical groups like TMAs and FFPE, thereby allowing the reproducible identification of spatial metabolite and protein signatures on many patients.
4	Sans M et al., 2017 [144]	DESI ambient imaging of serous ovarian tumor tissues for metabolic marker identification and the prediction of aggressiveness.	Identified the lipid and metabolite markers that differentiate tumor subtypes and predicted aggressiveness; this showed ambient MS as a fast clinically relevant metabolite mapping tool.

### 3.19. Year 2018

**Figure 20:**
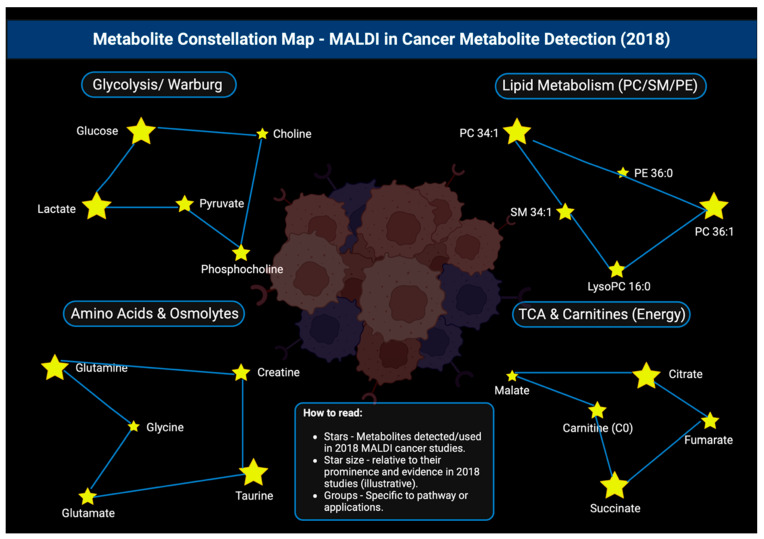
Constellation map showing the various metabolites that were detected by MALDI and studied in 2018, all arranged in their key metabolic pathways [151].

2018 saw practical method papers and reviews standardize the use of MALDI imaging of N-glycans in cancer tissues, which could be fresh-frozen or FFPE, clarifying PNGase F on-tissue release, matrix choices, and Orbitrap and FT-ICR readouts, which was key for the expansion beyond lipids to broader metabolite classes [146]. The year 2018 also marked a move from the development of methods to more clinically relevant applications of MALDI in detecting cancer metabolites [Figure 20]. MALDI imaging performed on breast cancer fTMA groups saw adenylate energy charges like ATP, ADP, and AMP and showed higher energy charges in tumors compared to non-tumors. This was an early example of spatial tumor bioenergetics readouts that were directly from patient samples [147]. Through colorectal-cancer tumor organoids, MALDI traced irinotecan and its metabolites, including SN-38 and SN-38G, over time and concentration, which showed non-co-localized drug versus active metabolite pools. This was evidence that MALDI imaging could read drug metabolism in situ for precision-oncology processes [148]. Table 19 shows the notable research from the year 2018.

**Table 19:** Notable research from year 2018.

No.	Author	Application	Result
1	Torata N et al., 2018 [147]	MALDI imaging of adenylate nucleotides like ATP, ADP, and AMP to compute energy charge in breast carcinoma compared to surrounding normal tissue.	Tumor regions had shown significantly higher energy charge and adenylate pool, which supported on-tissue mapping of tumor bioenergetics as a diagnostic tool.
2	Liu X et al., 2018 [148]	MALDI imaging performed on patient-derived colorectal cancer organoids to track irinotecan (CPT-11) and active or inactive metabolites during treatment response studies.	Able to spatially resolve drug metabolite patterns in organoids, showed heterogenous biotransformation like SN-38 and SN-38G, proving MALDI imaging as a preclinical pharmacometabolomics.
3	Sugihara Y et al., 2018 [149]	MALDI MSI of endogenous small metabolites in human malignant melanoma to map metabolic heterogeneity on tumor regions.	Showed distinct metabolic signatures that separated tumor sub-compartments and tumor vs. peri-tumoral skin, thus underscoring metabolic spatial heterogeneity in melanoma.
4	Drake RR et al., 2018 [150]	Developed an on-tissue MALDI MSI process for the detection of N-linked glycans from fresh-frozen and FFPE cancer tissues through enzymatic release and chemical stabilization.	Allowed the robust and reproducible imaging of more than 100 N-glycan species, notably increasing small metabolite coverage on cancer tissues. This improved sensitivity, spatial mapping, and compatibility with archived FFPE tumor samples.

### 3.20. Year 2019

**Figure 21:**
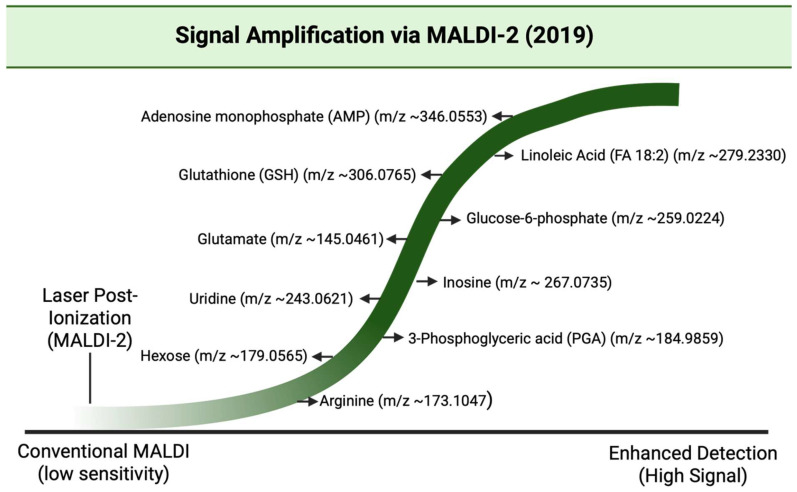
Wave diagram illustrating the enhanced detection of low-abundance metabolites using MALDI-2 (laser post-ionization) as reported by Barré et al., 2019 [25,156].

2019 had many advances, particularly in sensitivity, reproducibility, and clinical relevance in MALDI-based cancer metabolite detection. MALDI-2, which is the laser-induced post ionization variant of the MALDI family, was shown to significantly increase ion yields for drugs, lipids, and other small molecules, which improved detection limits and enabled the visualization of species that were previously unseen in MALDI detection [25] [Figure 21]. Work was also done to make MSI data analysis more accessible and reproducible, like in Galaxy-based MSI, which would lower the barriers for robust metabolite identification and inter-lab comparison in cancer studies. Shareable analysis methods also increased the confidence in metabolite biomarker findings [152]. High-resolution MALDI-MSI studies in 2019 had identified specific phosphoinositide (PI) compositions and other metabolite patterns that correlate with invasion and nodal metastasis, for example, in breast cancer. This strengthened the connection between spatial metabolite maps and clinical phenotypes [153]. Table 20 shows the notable research from the year 2019.

**Table 20:** Notable research from year 2019.

No.	Author	Application	Result
1	Barré FPY et al., 2019 [25]	The first broad demonstration of laser post ionization (MALDI-2) for imaging small molecules and drugs in tissue.	Hugely increased ion yields, improving the limits of detection, and allowing the visualization of metabolites and pharmaceuticals that were previously undetectable by normal MALDI.
2	Föll MC et al., 2019 [152]	Published the Galaxy MSI framework and training material to make MSI data analysis accessible and reproducible.	Gave open and shareable guidelines for MSI data preprocessing, normalization, segmentation and statistics which raised reproducibility and allowed for metabolite biomarker identification across labs.
3	Sun N et al., 2019 [154]	High-resolution MALDI MSI to map steroid sulfates in adrenocortical carcinoma (ACC).	Found steroid sulfation patterns that can classify prognosis in ACC, proving that MALDI can detect clinically relevant small molecules like steroids metabolic reprogramming in tumors.
4	Mittal P et al., 2019 [155]	MALDI MSI performed on primary cancer spheroids and organic models to look after its drug accumulation, metabolism and response.	Showed the spatial tracking of drug uptake and metabolites in 3-D patient-derived spheroids, thereby accepting MALDI-MSI as a preclinical pharmacometabolomics tool for therapy testing.

### 3.21. Year 2020

**Figure 22:**
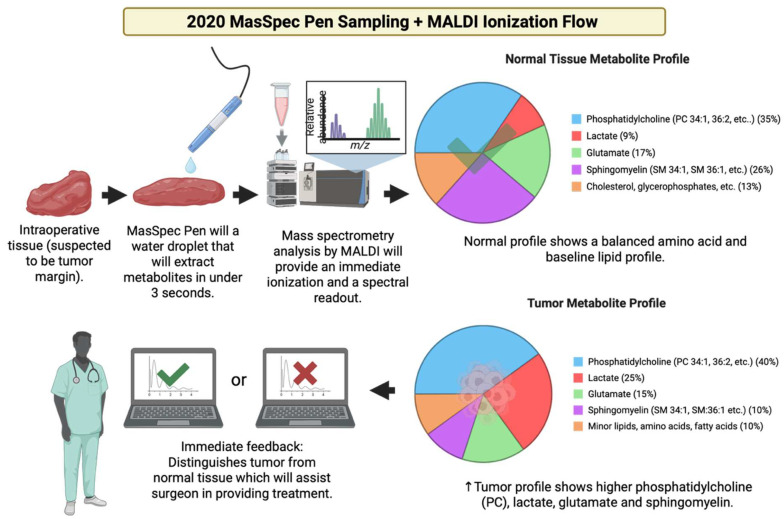
The MasSpec Pen was introduced in 2020 for intraoperative cancer metabolite detection which could be used in conjunction with MALDI by providing the sample extracted for further analysis [163].

2020 marked the integration of the MasSpec Pen into robotic surgery, namely into the da Vinci system and broader intraoperative processes that showed that the device can collect metabolic fingerprints in vivo without damaging tissue, which became a huge step towards metabolite-guided surgical decision support [157] [Figure 22]. The implementation and adaptations of MALDI-2 or plasma or post-ionization on commercial MSI platforms like timsTOF fleX and other atmospheric pressure implementations showed significantly increased ion yields for lipids, drugs, and metabolites and enabled the detection of species that were previously below MALDI limits. As a result, this materially improved small molecule and metabolite coverage in tissue [158]. The year 2020 also saw various widely accepted processes that enabled MALDI-MSI, including metabolites and lipids, whole exome, and RNA sequence from the same FFPE sections or other closely matched serial sections, which improved how spatial metabolite maps can be integrated with molecular pathology and retrospective clinical groups [27]. Table 21 shows the notable research from the year 2020.

**Table 21:** Notable research from year 2020.

No.	Author	Application	Result
1	Ščupáková K et al., 2020 [159]	Morphometric single-cell classification by using MALDI-MSI, combining image-based cell morphometrics with MALDI molecular maps.	Showed cell-level molecular classification on tissue section which advanced the near-single-cell metabolite lipid mapping in cancer tissue.
2	Andersen MK et al., 2020 [160]	MALDI-MSI performed on prostate tissue to simultaneously detect zinc and zinc-related small metabolites.	Was able to visualize zinc and its pathway metabolites in situ. This gave new metabolite markers related to prostate cancer biology.
3	Randall EC et al., 2020 [161]	MALDI-TOF MSI mapping of glioblastoma vs. nearby tissue metabolites.	Found the spatial shifts in metabolites like antioxidants, purine, and pyrimidine intermediates, 2-HG, lactate, glutamine, and citrate that had delineated tumor borders and glioblastoma subtypes.
4	Sun C et al., 2020 [162]	MALDI profiling of carnitine metabolism in breast cancer.	Were able to map 17 carnitine species including L-carnitine and acyl-carnitine in tumor tissue vs. normal tissue. Carnitine metabolism dysregulation was an excellent classifier with around 96% accuracy for breast cancer.

### 3.22. Year 2021

**Figure 23:**
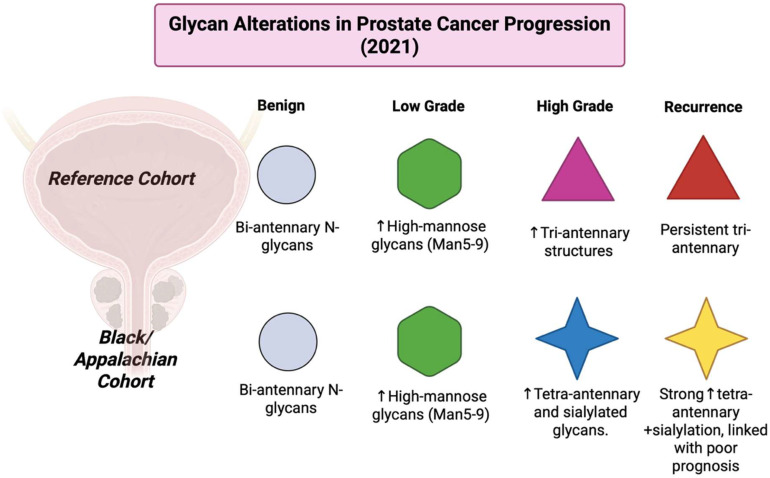
Timeline arc displaying the stages from benign tissue through low-grade, high-grade, and recurrent prostate cancer [169]. The arrow mean increase.

The year 2021 was mostly about translation and refinement, where studies grew larger, techniques used became more standardized, and the clinical usage of MALDI-MSI in detecting cancer metabolites became more advanced. The on-tissue N-glycan imaging, which is PNGase-F release, and MALDI-MSI were applied to large prostate cancer TMAs and was shown to identify N-glycan signatures that associate with tumor grade and recurrence, including the differences in patient groups [Figure 23]. This projected glycan imaging as an advanced spatial metabolite readout in cancer studies [164]. A few large MALDI studies were able to map metabolite patterns that correlated with the tumor genotype and malignant potential, for example, work conducted in pheochromocytoma and paraganglioma (PPGL) found kynurenine pathway and other metabolite alterations associated with metastatic behavior. These studies showed that MSI can reveal clinically useful metabolic reprogramming connected to tumor biology [165]. MALDI had also been applied to multicellular tumor spheroids by 3D in vitro models to explain how drugs, for example hydroxychloroquine, can change spatial metabolite distributions inside the spheroids. This strongly helped preclinical pharmacometabolomics processes that connect metabolic changes with treatment effects [166]. Table 22 shows the notable research from the year 2021.

**Table 22:** Notable research from year 2021.

No.	Author	Application	Result
1	Murakami M et al., 2021 [165]	MALDI MSI metabolomics of pheochromocytoma and paraglioma (PPGL) tissues across genotypes.	Showed tumor-genotype-related metabolic patterns and kynurenine pathway alternations associated with metastatic potential.
2	Chen Y et al., 2021 [166]	MALDI imaging performed on multicellular tumor spheroids to assess hydroxychloroquine response.	Traced the lipid metabolite changes like in acylcarnitine and PLs within spheroid zones, showing the usage of pharmacometabolomics.
3	Sun N et al., 2021 [167]	MALDI-MSI of native glycan fragments in PDAC FFPE tissues.	Found that specific native glycan fragments are independent prognostic factors in PDAC, thus stating glycan metabolites as tissue biomarkers.
4	Denti V et al., 2021 [168]	MALDI imaging spatial lipidomics performed on colorectal cancer linked to tumor infiltrating lymphocytes (TILs)	Discovered some lipid and metabolite features associated with immune infiltration; this was a step toward spatial immunometabolism profiling in cancer tissue.

### 3.23. Year 2022

**Figure 24:**
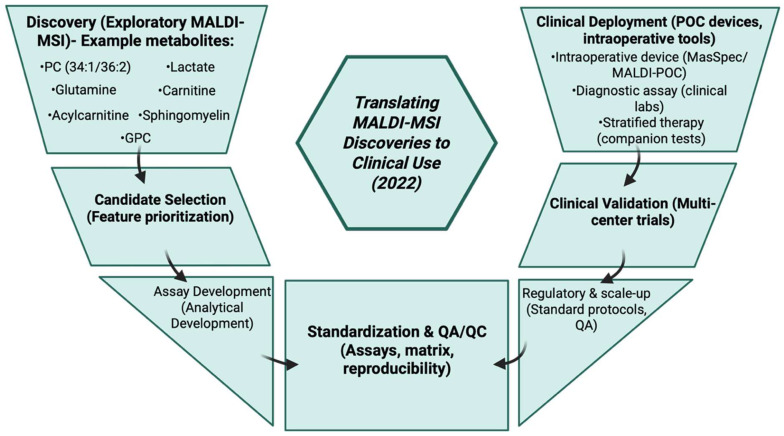
Dual funnel showing how MALDI metabolite discoveries (**left**) are refined and standardized to allow for clinical deployment (**right**) [177].

In 2022, MALDI metabolomics really moved into clinical-scale and FFPE-related processes [Figure 24]. A pathology-compatible process was able to align MALDI-MSI small metabolite maps from FFPE sections with the whole exome and RNA sequence; this showed that spatial metabolite features can be co-registered with mutations and expression in head- and neck-related cancer. This validated MALDI metabolite detection in routine histopathology frameworks [170]. Ultra-high-resolution MALDI-FT-ICR MSI was conducted on 782 renal carcinomas and was able to identify pathway-level metabolite features that were associated with overall survival and subtype-specific prognosis like ccRCC, pRCC, and chRCC, which all pushed MALDI metabolomics from just diagnosis to outcome prediction [171]. A multiplatform framework was able to combine MALDI metabolite imaging with nuclear magnetic resonance (NMR) to cross-validate small molecule assignments in breast cancer xenografts and in other cancers, which was proof that orthogonal modalities can boost up the identification of metabolites [172]. Table 23 shows the notable research from the year 2022.

**Table 23:** Notable research from year 2022.

No.	Author	Application	Result
1	Bollwein C et al., 2022 [173]	MALDI-MSI proteomic and peptide profiling to differentiate pancreatic ductal adenocarcinoma (PDAC) vs. cholangiocarcinoma.	Achieved an impressive ~95% classification accuracy on a pixel-by-pixel level by using MALDI-MSI-derived peptide features with machine learning classifiers such as gradient boosting (GB), support vector machine (SVM), and k-nearest neighbors (KNN) on formalin-fixed paraffin-embedded (FFPE) tissue microarrays.
2	Li Z et al., 2023 [174]	High-throughput serum metabolite detection through MALDI-MS together with machine learning.	Was able to identify 13 distinct features that were significantly different (*p* < 0.001) between lung cancer patients and healthy specimens; 6 of these were identified as intact metabolites.
3	Han Y et al., 2022 [175]	Adapted on-tissues derivatization with MALDI-MSI for the isomer specific imaging of monosaccharides.	Allowed for the differentiation and relative quantification of monosaccharide isomers directly in tissue sections using MALDI, thus improving the specificity of carbohydrate-based metabolite imaging.
4	Sommella E et al., 2022 [176]	MALDI metabolite imaging of parotid gland tumors.	Was able to identify distinct metabolomic and lipidomic spatial signatures that differentiate tumor vs. surrounding healthy/parenchymal regions.

### 3.24. Year 2023

**Figure 25:**
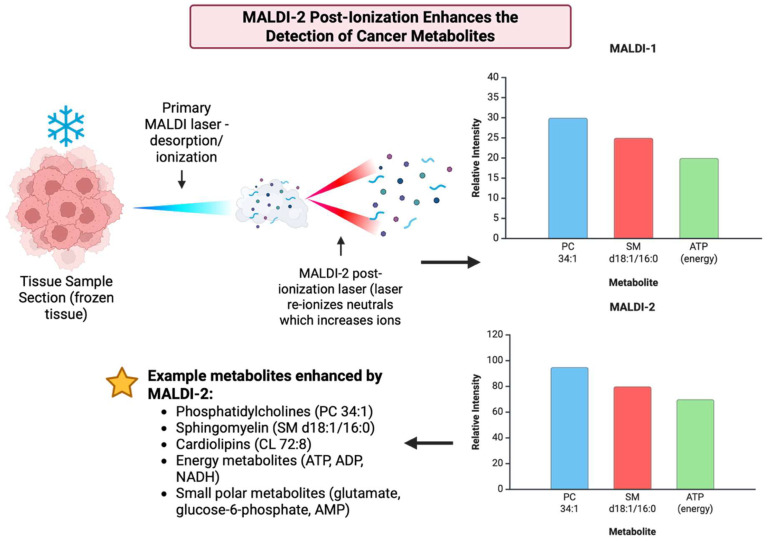
The additional laser by MALDI-2 selectively ionizes neutrals, increasing the coverage of phospholipids, sphingolipids, and energy metabolites in comparison to conventional MALDI [184].

In 2023, studies showed that MALDI-2 was able to reveal tumor-specific low-MW metabolites that were not seen with conventional MALDI and increased the signal intensity to up to around 20 times for some metabolites and thus significantly improved the detection of cancer-related small molecules [26] [Figure 25]. Systematic solvent wash protocols, for example, MeOH and 0.05% FA, were able to remove ion-suppressing lipids and proteins while still being able to preserve localization. This boosted the detection of phosphate-containing energy metabolites like ATP, ADP, and AMP and allowed spatial isotope tracing, also called iso-imaging of metabolite flux in organs, and was seen to be important in cancer metabolite mapping [178]. Reviews and experimental studies showed optimized matrix choices and new matrix materials, including nanoparticle-based matrices and atmospheric-pressure MALDI developments, which boosted the ionization efficiency for metabolites and lipids that were associated with cancer [179]. Table 24 shows the notable research from the year 2023.

**Table 24:** Notable research from year 2023.

No.	Author	Application	Result
1	Schwaiger-Haber M et al., 2023 [180]	MALDI and DESI-MSI was combined with a stable isotope labelling in a GL261 glioma mouse model.	Was able to map spatial distributions of metabolite abundances and fluxes derived from isotopes on glioma vs. normal brain; it showed a 3× increase in de novo fatty acid synthesis flux and 8× increase in fatty acid elongation in tumor tissue.
2	Stopka SA et al., 2023 [181]	Developed a tissue mimetic calibration array (Chemical QuantArray) that was placed on MALDI slides to quantify endogenous metabolites in tissue sections.	Gave a practical calibration approach by using isotopically labeled standards on the same slide. This improved the precision of MALDI quantification on multiple metabolites, which was a step toward clinically relevant metabolite measurements.
3	Ma B et al., 2023 [182]	MALDI profiling of xenograft models and 21 human hepatocellular carcinoma (HCC) tissues.	Spatially resolved metabolic profiling identified metabolites that were altered during HCC development and validated many metabolic markers in patient sample, showing the importance of metabolic reprogramming in HCC.
4	Wangyan T et al., 2023 [183]	Deep-learning (mNet) framework was used to analyze MALDI tissue microarrays from breast and lung cancer groups.	Improved tissue classification and preserved the spatial contexts vs. point-level methods. It proved that ML can help with high-throughput MALDI cancer tissue analysis.

### 3.25. Year 2024

Several reports in 2024 had refined matrix selection, solvent washes, and MALDI-2 frameworks specifically for small polar metabolites and isotope tracing experiments, thereby able to produce higher coverage and stronger signals for metabolites, including steroids, small polar organic acids, and phosphorylated species in tumor tissues. This had popularized MALDI-2 even more but also showcased its usability in practical, tissue-focused processes [185]. New mass-guided single-cell MALDI procedures, for example, PRISM-MS and its related methods, had allowed for the analysis of low-mass, hydrophilic metabolites at the single-cell or near-single cell level, which was important for studying metabolic heterogeneity within tumors and their microenvironment. These methods had also included cell-targeting strategies and metabolome-preserving sample handling [186]. Frameworks were introduced to align MALDI-MSI metabolite maps with orthogonal methods such as imaging mass cytometry and spatial transcriptomics to allow for the cell-type-resolved metabolic readouts in tumor sections and to connect metabolic phenotypes to cellular identity and immune contexture [187]. Table 25 shows the notable research from the year 2024.

**Table 25:** Notable research from the year 2024.

No.	Author	Application	Result
1	Bharti A et al., 2024 [188]	Combination of MALDI-MSI with two photon laser scanning microscopy and histology on human colorectal cancer tissue section.	Showed local heterogeneity in colorectal cancer by co-registering MALDI molecular maps with collagen structure and 2-photon features that highlighted region-specific molecular signatures that are linked to tumor microenvironment [Figure 26].
2	Stillger MN et al., 2024 [189]	Review on MALDI-MSI use on rare cancers like sarcoma.	Showed discoveries like metabolic signatures by MALDI in sarcomas and put focus on the challenges in sample prep and FFPE usage and proposed processes to translate MALDI spatial biomarkers for rare cancer research.
3	Brorsen LF et al., 2024 [190]	MALDI characterization of cutaneous squamous cell carcinoma (SCC) along with machine learning.	Showed MALDI-MSI metabolites features that distinguish SCC tissue regions and support automated histology-aligned classifications.
4	Hartig JP et al., 2024 [191]	MALDI-MSI analysis of prostate cancer tissue microarrays, putting focus on N-glycan ECM protein composition.	Were able to identify N-glycan and collagen ECM protein patterns that differentiated the outcome groups and suggested ECM linked molecular features as a probable early predictor of prostate cancer metastasis.

**Figure 26:**
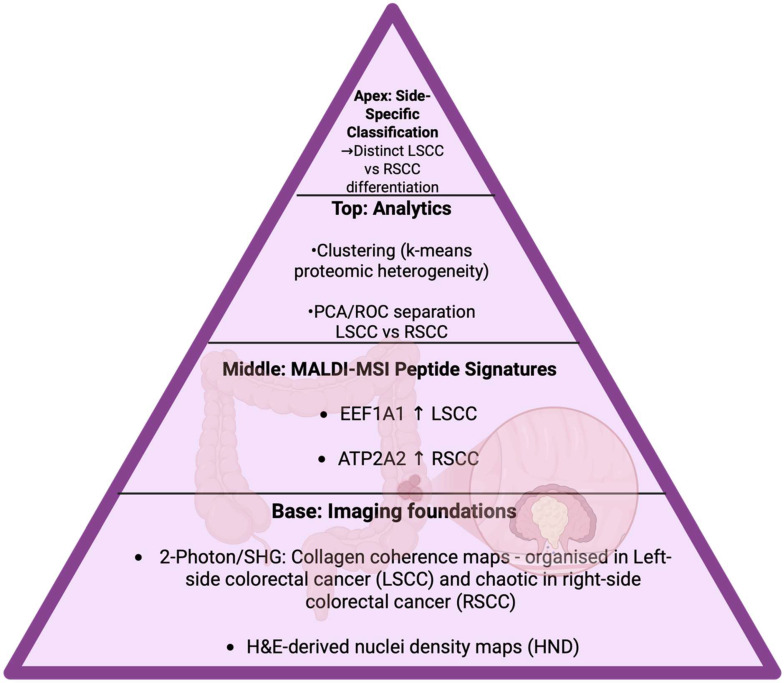
The pyramid summarizes Bharti et al., 2024 [188], where tri-modal co-registered imaging was integrated. Peptide signals assigned to EEF1A1 were enriched in LSCC while ATP2A2 signals were enriched in RSCC [192]. The arrow mean the increase.

### 3.26. Year 2025

**Figure 27:**
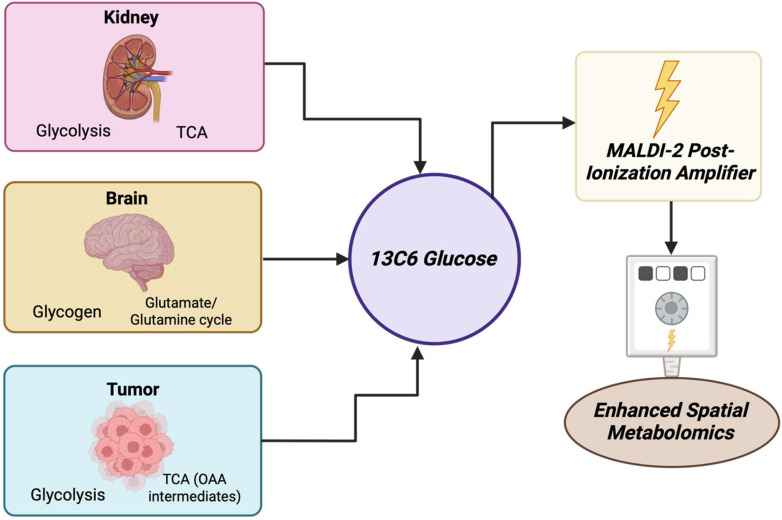
Isotope circuit-board showing 13C6 Glucose tracer distributing to kidney, brain, and tumor “boards”. MALDI-2 acts as a post-ionization amplifier that amplifies weak ions to enable further enhanced metabolomics [200].

2025 has shown new sample pretreatment validation steps that improved the sensitivity and strength of MALDI-MSI for polar and 2H-labeled metabolites, which was a step toward capturing cancer-relevant small molecules that were usually hard to ionize and retain spatially [193]. Figure 27 shows MALDI-2 which acts as a post-ionization amplifier that amplifies weak ions to enable further enhanced metabolomics; MALDI-MSI was used to visualize L-arginine and its related metabolites in tumors during its treatment with the dual arginase inhibitor OATD-02, thus linking the spatial metabolite changes to enhanced anti-PD-efficacy, which was proof that MALDI metabolite maps were able to quantify-on target pharmacodynamics in the tumor microenvironment [194]. A 2025 procedure had specifically addressed the sectioning and preparation of lung cancer tissue for MSI along with parallel microscopy to guide ROIs, and it helped labs reproducibly capture metabolite distributions in delicate-necrotic or post-treatment tumor areas [195]. Table 26 shows the notable research from the year 2025.

**Table 26:** Notable research from year 2025.

No.	Author	Application	Result
1	Phulara NR et al., 2025 [196]	Mouse pancreatic ductal adenocarcinoma (PDAC) tumors treated with gemcitabine for the MALDI imaging of lipid metabolites.	Gemcitabine shifted tumor phosphatidylcholine metabolism and PCs such as 30:0, 32:3, 34:2, 36:1, 36:2 had been elevated in treated tumors and indicated therapy linked remodeling.
2	Chen B et al., 2025 [197]	The evaluation of sphingolipid detection by MALDI which was validated on PyMT breast cancer tissue and glioblastoma samples.	Put forth the best-practice matrices and ion modes, clarified fragmentation artifacts, for example, SM into S1P/C1P and showed the validated detection of ceramides and glycosphingolipids in cancer
3	Chen Y et al., 2025 [198]	Optimized MALDI-2 MSI to be able to trace stable isotopes in mouse breast, kidney and brain tumors to map ^13C-glucose derived intermediates.	Enabled the spatial glycomics of multiple pathways such as PPP, glycolysis, and TCA in tumors which revealed heterogeneity and markedly expanding low-MW metabolite coverage.
4	Krestensen KK et al., 2025 [199]	GBM patient-derived single cells and combined with high-resolution MALDI-MSI with MALDI-IHC markers.	The multimodal single-cell procedure showed cell-type-specific lipid profiles for example, PCs, TGs, and SMs and was able to accurately classify GBM vs. neuronal cells and showed metabolite-informed phenotyping.

## 4. Recent Trends and Future Directions

### 4.1. Artificial Intelligence and Deep Learning

Recent trends and advances have shown that integrating artificial intelligence and deep learning with MALDI-MS imaging has transformed cancer metabolite detection. Machine learning models are now able to classify tumor vs. normal tissue and cancer subtypes directly from MALDI metabolite maps, as demonstrated in skin cancers such as basal cell carcinoma and cutaneous squamous cell carcinoma [190]. Deep learning frameworks such as mNet, massNet, and msiPL have outperformed regular peak-based analysis by extracting non-linear features from high-dimensional spectra, which allowed for a more accurate diagnosis and biomarker discovery [183]. At the same time, region-specific segmentation models were able to isolate heterogenous tumor niches in breast cancer tissue, while ML-assisted annotation tools such as METASPACE-ML had improved confidence during metabolite identification [201]. All these developments in integrating AI and deep learning with MALDI-MSI have made cancer metabolomics faster, more accurate, and clinically translatable by connecting raw spectra to diagnostic and biological insights.

### 4.2. Personalized Medicine

MALDI-MSI has been increasingly developing personalized oncology by being able to generate spatially resolved metabolic profiles directly from individual tumor biopsies. For example, when patient-derived organoids were processed via MALDI-MSI, they showed drug metabolism patterns and metabolic vulnerabilities that were unique to each patient, which helped in deciding customized therapy for patients [202]. In clear-cell renal carcinoma, MALDI-MSI metabolic signatures such as elevated cyclic GMP and glutathione metabolism were able to correlate with the prognosis of patients, showing their potential for personalized prognostic stratification [203]. Importantly, rapid MALDI-MSI procedures have been demonstrated for intraoperative uses, thereby suggesting their feasibility in real-time, patient-specific margin assessment conducted during surgery [204]. Looking forward to the future, the integration of MALDI spatial metabolomics with multi-omics layers using artificial intelligence would shine light on individual tumor metabolic heterogeneity and be able to guide precision therapies.

### 4.3. Regulatory Outlook

Regulatory progress for MALDI-based cancer metabolite detection has shifted from just exploratory research to now towards its clinical translation, but big challenges lie ahead. Prerequisites such as a standardized sample preparation technique and rigorous analytical validation, including precision, linearity, and matrix effects, are being emphasized as necessary for its clinical use [205]. Recent literature has highlighted the need for more multicenter reproducibility studies and powerful clinical validation groups to show its clinical utility and meet the FDA’s evidentiary standards for diagnostics and companion diagnostics [206]. Mixed opinions regarding US policies, particularly over the VALID Act, and a much tighter oversight of laboratory-developed tests (LDT) could speed up formal regulatory review but may also raise barriers for its academic LDT usage. Spatial metabolomics such as MALDI-MSI adds strong diagnostic value but also makes it complex for regulatory acceptance because such spatial procedures require image-analytic validation and interoperability standards [207] The field overall has a near-future direction that is moving toward standardized operating procedures, collaborative clinical trials, and an early discussion with regulators to streamline the pathway from discovery to FDA-cleared or CLIA-compliant tests [205].

### 4.4. Limitations and Challenges

Advances in recent MALDI-based metabolite detection have improved the spatial mapping of cancer metabolism, but important challenges still remain. Challenges include the current matrix interface, which does not allow for the detection of small polar metabolites as the organic matrix produces overlapping peaks [208], the suppression of ions, and the variability of sample preparation, which often decreases sensitivity, resulting in the restriction of single-cell applications [209]. It is difficult to have reliable metabolite identification due to the isobaric and isomeric species requiring high-resolution MS or on-tissue MS/MS [210]. Moreover, MALDI is unable to well-ionize highly polar or volatile metabolites, as that needs derivatization or alternative matrices. The lack of a standardized preprocessing and analysis pipeline in the field makes data volume and complexity additional barriers [211]. For translation, hurdles such as clinical validation, regulatory approval, and cost of instruments remain and prevent its routine clinical adoption. Addressing these issues and challenges with better matrices, standardized workflows, highly advanced computational tools, and multi-omics integration will be crucial for its clinical impact in the future.

Matrices used in MALDI-based cancer metabolite detection are often small aromatic compounds that generate a significant number of low m/z ions. These ions can overlap or obscure the signals of endogenous small metabolites such as amino acids, nucleotides, and fatty acids. This effect is called matrix noise and leads to results such as the loss of specificity as genuine metabolic biomarkers become difficult to distinguish from background peaks and signal interference particularly near matrix cluster regions where matrix-derived ions will disguise metabolite peaks. It can be overcome with strategies such as the use of alternative matrices like gold nanoparticles and graphene oxide which will minimize the interference of the organic matrix, and the generation of cleaner spectra by the use of matrices with lower fragmentation or weaker background like 9-aminoacridine.

Issues surrounding quantitative reliability include the ion suppression varying from pixel-to-pixel extraction and efficiency differing across tissues. The use of on-tissue internal standards can normalize local ionization and extraction whereas targeted modes can increase precision. MALDI’s small molecule coverage also remains an issue due to the polar metabolites showing poor ionization, classic UV matrices promoting bias detection and insource fragmentation that complicates identification. On-tissue chemical derivatization strategies can label functional groups that will boost ionization and shift the *m*/*z* value away from interferences. The use of alternative matrices combined with tuned laser fluence can also help in improving small molecule coverage issues. Another challenge is MALDI’s data complexity regarding the large size of the datasets, the essential registration to histology, and sparse spectra, which can be overcome by the use of spatial cross validation and leakage checks in modernized machine learning. More reproducible workflows can also help reduce the data complexity by the use of containerized analysis along with the pre-registration of analysis plans for clinical studies. Regulatory readiness also remains a concern as MALDI is not yet a routine regulated diagnostic as its analytical validation and clinical performance need to be measured before moving into the in vitro diagnostic (IVD) framework. Steps such as fixing the tissue type, pre-analytical steps, acquisition parameters, and matrix-reagent lock controls permanently in the assay design, would improve the regulatory readiness of MALDI.

## 5. Conclusions

Over the past 25 years, the detection of cancer metabolites by MALDI has evolved from just a proof-of-concept experiment to a useful and powerful platform in the world of spatial metabolomics and translational oncology. Early studies were able to demonstrate MALDI-MSI’s ability to map metabolites with high mass range and spatial resolution, which allowed the visualization of lipids, small molecules, and proteins within the tissue sections with resolutions that were down to a few micrometers. Breakthroughs in prostate cancer research were able to show MALDI-MSI’s capacity to differentiate malignant tissue from benign tissue through altered distributions of biomarkers such as MEKK2 fragments and phosphatidylinositol, which achieved sensitivities and specificities of more than 85% [212]. Further advancements also include the simultaneous spatial detection of prostate-specific metabolites such as citrate, zinc, and aspartate, thus providing a metabolite signature with diagnostic and prognostic potential.

Technological innovations have steadily advanced MALDI’s metabolite coverage and sensitivity. The introduction of MALDI-2 (laser-post ionization) was able to almost double the number of detectable on-tissue mass features and revealed tumor-specific metabolites that could not be detected previously using conventional MALDI [26]. At the same time, the integrative approaches that linked MALDI-MS with machine learning had enabled the identification of serum metabolites that could distinguish lung cancer from healthy controls with a high statistical significance [13].

Recent detailed reviews have pointed out the expanding role of MALDI-MSI in discovering metabolic reprogramming in cancer and in the integration of AI, multi-modal imaging, and three-dimensional spatial metabolomics [11]. All these developments emphasize MALDI’s progression as a robust tool for in situ cancer metabolite detection and its increasing translational relevance. Although challenges still remain, particularly around its standardization, reproducibility, and multi-center validation, MALDI-MSI’s growing analytical sophistication and biomarker potential put it in a strong position to advance in precision oncology in the coming years.

## Figures and Tables

**Table 1:** Notable research from year 2000.

No.	Author	Application	Result
1	Kussmann M, Roepstorff P., 2000 [21]	Step-by-step sample preparation processes for analyzing peptides and proteins by MALDI-MS.	It gave reproducible and refined sample preparation methods that improved MALDI signal quality and reproducibility. It gave instructions on when to use certain deposition methods and matrix choices.
2	Palmer-Toy DE et al., 2000 [35]	Direct MALDI-TOF MS profiling from laser captured micro-dissected (LCM) breast cancer and normal epithelial cells.	Showed distinct protein and peptide spectral patterns from 1250 cell micro-dissected regions which proved the feasibility of MALDI on histology defined microdomains in cancer tissue.

**Table 2:** Notable research from year 2001.

No.	Author	Application	Result
1	Stoeckli M et al., 2001 [36]	MALDI imaging mass spectrometry of tissue sections with a glioblastoma example.	Showed spatially resolved protein/peptide ion maps across proliferating vs. necrotic tumor areas and popularized MALDI for molecular histology in cancer.
2	Todd PJ et al., 2001 [37]	Comparative paper on organic ion imaging methods like TOF-SIMS and MALDI for biological tissue.	Described how SIMS and MALDI could produce molecular images from tissues and compared their strengths (SIMS-higher spatial resolution, MALDI-broader mass range).
3	Chaurand P et al., 2001 [40]	MALDI profiling of azoxymethane-induced mouse colon tumors.	Identified tumor-specific protein signals from matched normal/tumor colon tissue. Showed MALDI tissue profiling for cancer metabolite discovery.
4	Le Naour F et al., 2001 [42]	2-D PAGE and MALDI-TOF to identify tumor antigens, proteins in breast cancer serum, and tumor cell lines.	Identified RS/DJ-1 as a new circulating tumor antigen in breast cancer which showed MALDI’s role in biomarker detection from clinical samples.
